# The fungal metabolite chaetocin is a sensitizer for pro-apoptotic therapies in glioblastoma

**DOI:** 10.1038/s41419-019-2107-y

**Published:** 2019-11-26

**Authors:** Ezgi Ozyerli-Goknar, Ilknur Sur-Erdem, Fidan Seker, Ahmet Cingöz, Alisan Kayabolen, Zeynep Kahya-Yesil, Fırat Uyulur, Melike Gezen, Nazife Tolay, Batu Erman, Mehmet Gönen, James Dunford, Udo Oppermann, Tugba Bagci-Onder

**Affiliations:** 1 Brain Cancer Research and Therapy Laboratory, Koç University School of Medicine, 34450 Istanbul, Turkey; 2 Department of Computational Biology, Koç University, 34450 Istanbul, Turkey; 3 Molecular Biology, Genetics and Bioengineering Program, Faculty of Engineering and Natural Sciences, Sabanci University, Istanbul, Turkey; 4 Department of Industrial Engineering, College of Engineering, Koç University, İstanbul, Turkey; 5 Botnar Research Centre, NIHR Biomedical Research Centre Oxford, University of Oxford, Oxford, OX3 7LD UK; 6 Structural Genomics Consortium, University of Oxford, Oxford, OX3 7DQ UK; 7 FRIAS, Freiburg Institute of Advanced Studies, University of Freiburg, 79104 Freiburg, Germany

**Keywords:** CNS cancer, Apoptosis

## Abstract

Glioblastoma Multiforme (GBM) is the most common and aggressive primary brain tumor. Despite recent developments in surgery, chemo- and radio-therapy, a currently poor prognosis of GBM patients highlights an urgent need for novel treatment strategies. TRAIL (TNF Related Apoptosis Inducing Ligand) is a potent anti-cancer agent that can induce apoptosis selectively in cancer cells. GBM cells frequently develop resistance to TRAIL which renders clinical application of TRAIL therapeutics inefficient. In this study, we undertook a chemical screening approach using a library of epigenetic modifier drugs to identify compounds that could augment TRAIL response. We identified the fungal metabolite chaetocin, an inhibitor of histone methyl transferase SUV39H1, as a novel TRAIL sensitizer. Combining low subtoxic doses of chaetocin and TRAIL resulted in very potent and rapid apoptosis of GBM cells. Chaetocin also effectively sensitized GBM cells to further pro-apoptotic agents, such as FasL and BH3 mimetics. Chaetocin mediated apoptosis sensitization was achieved through ROS generation and consequent DNA damage induction that involved P53 activity. Chaetocin induced transcriptomic changes showed induction of antioxidant defense mechanisms and DNA damage response pathways. Heme Oxygenase 1 (*HMOX1*) was among the top upregulated genes, whose induction was ROS-dependent and HMOX1 depletion enhanced chaetocin mediated TRAIL sensitization. Finally, chaetocin and TRAIL combination treatment revealed efficacy in vivo. Taken together, our results provide a novel role for chaetocin as an apoptosis priming agent and its combination with pro-apoptotic therapies might offer new therapeutic approaches for GBMs.

## Introduction

Glioblastoma multiforme (GBM) is grade IV glioma^1^ that represents >45% of primary brain tumors with an incidence of 3.2/100,000 per year^2^. As standard of care, surgery with maximal tumor resection followed by external‑beam radiation with concomitant Temozolomide therapy is applied. Despite recent therapeutic developments^3^, the median survival for GBM patients is 14.6 months, and only a small fraction of patients (3%) live longer than 5 years after diagnosis^4,5^ highlighting an unmet need to develop alternative treatment strategies.

One therapeutic strategy is to selectively induce cancer cell death. Apoptosis is a programmed cellular self-destruction process^6^, which fosters the maintenance of tissue homeostasis through elimination of pre-malignant cells, therefore it is a barrier for cancer progression. Apoptosis can be triggered extrinsically with proapoptotic ligands, such as tumor necrosis factor-related apoptosis-inducing ligand (TRAIL)^7^ that utilize the death receptors (DR4/DR5), Death Inducing Signaling Complex (DISC) and initiator caspases (caspases 8 and 10)^8^. Intrinsic apoptosis is activated via DNA damaging drugs^9^ or other agents, such as BH3 mimetics^10^, which involves mitochondrial outer membrane permeabilization via BAK or BAX, consequent Cytochrome C release and effector caspase activation^11,12^. The link between extrinsic and intrinsic apoptosis is established by BID, which is truncated by active Casp8 and lead to oligomerization of BAK or BAX into mitochondrial pores.

Apoptosis induction by TRAIL is selective for cancer cells^13,14^. However, most cancer cell types, such as chronic lymphocytic leukemia^4^, medulloblastoma^15^, astrocytoma^16^, and glioma^17^ are resistant to TRAIL, which render clinical application of TRAIL inefficient. Resistance might stem from changes in the balance of pro-apototic and anti-apoptotic signal mediators, mutations, altered glycosylation, dysregulated endocytosis, reduced expression of death receptors, and overexpression of decoy receptors^18^.

Epigenetic modulation of death receptor mediated pathway has been a successful approach for increasing TRAIL efficacy on tumor cells. To this end, histone deacetylase (HDAC) inhibitors, such as MS275^19^, SAHA^20^, Valproic acid^21^, Depsipeptide^22^, SBHA^23^, LAQ824^24^ have been shown to augment TRAIL responses in various tumor types including prostate cancer, primary myeloid leukemia, melanoma, breast cancer, medulloblastoma, CLL, and GBM. Alteration of DNA methylation has also been proven to be effective in modulating TRAIL response of hepatoma, Burkitt’s lymphoma, SCLC cells as exemplified by 5-aza-2′-deoxycytidine treatment^15,25^, combination treatment of decitabine and Valproic acid^26^, treatment with DNMT1 inhibitor Iso-3^27^ and by DNMT1 and DNMT3b silencing^28^.

Epigenetic compounds that could change the expression of apoptosis-related machinery could be excellent secondary agents to augment TRAIL efficacy. However, to our knowledge, there has been no epigenetic drug-centered chemical screen conducted for GBM cells. Here, we undertook a chemical screening approach to identify novel TRAIL-sensitizers from a library of different epigenetic modifier chemical probes. Testing the efficacy of each individual drug alone and on potentiation of TRAIL response in GBM cells, the screen revealed the fungal metabolite chaetocin, previously identified as unselective histone methyltransferase inhibitor^29^ as a potential TRAIL-sensitizing agent. The apoptosis potentiating effects of chaetocin involved induction of ROS and modulation of stress response and DNA damage pathways in GBM cells.

## Materials and methods

### Cell culture

U87MG, U373, and T98G GBM cell lines were purchased from American Tissue Type Culture Collection (ATCC) and authenticated. U87MG-TR cells were TRAIL resistant derivatives of U87MG cells (manuscript under review). 293T cells were kind gift of Dr. Tamer Onder (Koç University, Turkey). Cells were grown in DMEM medium (Gibco, USA) supplemented with %10 fetal bovine serum (Gibco, USA) and %1 Pen/Strep (Gibco, USA) in humidified incubator at 37 °C with 5% CO_2_ level. Primary cell line GBM8 was obtained from Dr. Hiroaki Wakimoto (Massachusetts General Hospital, Boston, MA) and grown as neurospheres in cell culture flasks containing EF medium (Neurobasal medium supplemented with EGF, FGF, B-27, N2, Heparin, L-Glutamine, and Pen/Strep).

### Reagents

TRAIL was commercially supplied (SuperKiller, Enzo Life Sciences, Farmingdale, NY, USA) or produced from 293T cells as described^30^. Caspase inhibitors (BD Pharmingen, San Diego, CA, USA) were: Z-VAD-FMK (general caspase inhibitor), Z-FA-FMK (negative control). Bcl-2, Bcl-xL inhibitors ABT-263 and WEHI-539 were purchased from Cayman Chemicals (Ann Arbor, MI, USA). FasL and N-acetyl-L-cysteine (NAC) were purchased from Sigma-Aldrich (MO, USA). NUTLIN-3a was purchased from MedChemExpress (NJ, USA). Doxycycline was purchased from Sigma-Aldrich (Cat. No: D9891). D-luciferin was purchased from Biotium (CA, USA). Chaetocin was purchased from two sources (C9492–1mg, Sigma-Aldrich, MO, USA) and (S8068, Selleckchem, Houston, TX, USA). The epigenetic tool library was constructed as described^31^.

### Cell viability, caspase activity, and caspase inhibition assays

Cell viability was detected by ATP based Cell Titer-Glo (CTG) Luminescent Cell Viability Assay (Promega) according to the manufacturer’s instructions using a plate reader (BioTek’s Synergy H1, Winooski, VT, USA). 5000 cells/well were seeded to 96-well plates (Corning Costar, clear bottom black side) as triplicates for each condition and treated with corresponding chemicals of interest for defined period. For all cell viability assays, Chaetocin was applied simultaneously with apoptosis inducers (TRAIL/FasL/BH3 mimetics). For caspase inhibition assay, cells were subjected to Chaetocin treatment for 24 h prior to TRAIL treatment. Z-FA-FMK (Negative Control for Caspase Inhibitors) or Z-VAD-FMK (General Caspase Inhibitor) pretreatments were performed at 20 µM final concentration for 24 h before the following drug treatments. For caspase activity assays, cells were treated with chaetocin (100 nm, 24 h) followed by TRAIL (100 ng/ml for 3 h). Caspase 3/7 activity was measured by Caspase-Glo® 3/7 (Promega) assays according to manufacturer’s instructions. For measurement of caspase activity, cells were subjected to Chaetocin treatment for 24 h followed by TRAIL treatment for 3 h since caspase cleavage is evident at early stages of apoptosis. NAC was used as ROS scavenger. Cells were pretreated with NAC (10 μM) for 24 h. Next day cells were treated with the drug of interest in the presence of NAC and cell viability was measured.

### Live cell imaging

All live-cell imaging experiments were carried out by Olympus Xcellence Pro inverted microscope (Center Valley, PA, USA) with a ×10 air objective in a chamber at 37 °C, supplied with 5% CO_2_. Cells were seeded as 150.000 cells/well to 6-well plates and treated with chemicals of interest simultaneously in combination. Time-lapse images were captured right after drug treatments with 5 or 6 min intervals. From each well, random positions were recorded to obtain image stacks and death/live cells in each image were counted using the ImageJ Software (NIH Image, Bethesda, MD, USA). Quantifications were performed by counting 3 different image fields for each condition for selected time points.

### Quantitative RT-PCR

RNA isolation and cDNA synthesis were performed as described^32^. Primers used for qPCR are listed in Supplementary Table 1.

### Western blotting

Western blots were performed as described^32^. Cells were treated with Chaetocin for 24 h followed by 3 h TRAIL treatment to check caspase cleavage, Bid truncation and PARP cleavage. Western blots involving NAC-treated samples were performed on cells pretreated with NAC for 24 h followed by Chaetocin and TRAIL simultaneous combinatorial treatment for additional 24 h. All antibodies are listed in Supplementary Table 2.

### Annexin V/PI staining

Cells were seeded to 6-well plates (300,000 cells/well). After simultaneous treatment with chaetocin and TRAIL (100 nM and 100 ng/ml, respectively, for 24 h), all cells (both live cells attached to culture plate and dead cells free floating in medium) were harvested and pelleted. Pellets were washed in cold PBS, centrifuged and resuspended in 500 µl 1× Annexin binding buffer (1 × 10^6^ cell/ml). One hundred microliter of cell suspension was transferred to BD flow tubes and 5 µl of Alexa Fluor 488 Annexin V (ThermoFisher, Waltham, MA, USA) and 1 µl of 100 µg/ml PI working solution (5 µl of 1 mg/ml PI stock diluted in 45 µl Annexin binding buffer) were added. Cell suspension was incubated at room temperature for 15 min. Four hundred microliter Annexin V binding buffer was added. Stained cells were analyzed by BD Accuri C6 (BD Biosciences, USA) flow cytometer (excitation 488 nm, emission 530/575 nm) and 10,000 events were recorded for each sample.

### Terminal deoxynucleotidyl transferase dUTP nick end labeling (TUNEL) assay

Cells were seeded to 12-well plates (25,000 cells/well) on glass coverslips. Chaetocin and TRAIL simultaneous treatment (100 nM, 100 ng/ml, respectively) was performed for 24 h. After washing with PBS, air dried cells were fixed by 300 µl fixation solution (4% PFA in PBS, pH 7.4, freshly prepared) at 4 °C for 1 h. After rinsing 3 times with PBS, 300 µl Blocking solution (3% H_2_O_2_ in methanol) was added for 10 min at RT. Coverslips were rinsed with PBS 3 times and then incubated in 30 µl permeabilization solution (0.1% TritonX-100 in 0.1% sodium citrate, freshly prepared) at RT. After drying, 50 µl TUNEL reaction mixture (5 µl enzyme solution + 45 µl label solution) was added on top of each coverslips and samples were incubated 60 min at 37 °C. Coverslips were washed 3 times and incubated with DAPI dye, sealed and visualized by Leica DMi8 inverted microscope (Leica Microsystems, Germany). Quantification of images was done with ImageJ software (NIH Image, NIH Bethesda, USA).

### YO-PRO-1/PI staining

Cells were seeded to 12-well plates (30,000 cells/well). Chaetocin and TRAIL simultaneous treatments (100 nM, 100 ng/ml, respectively) were performed for 6 h. Wells were rinsed once with PBS followed by incubation in 300 µl staining solution (1 µM YO-PRO-1 by Invitrogen Cat. No: Y3603, Thermo Fisher, USA and 1:1000 PI (1 mg/ml) in PBS) for 15 min at 37 °C in dark. Each well was visualized, and representative images were taken by Nikon Eclipse TS100 Inverted Fluorescence Microscope (Nikon Instruments Inc., NY, USA). Quantification of images was done with ImageJ software (NIH Image, NIH, Bethesda, USA).

### RNA sequencing

Cells were seeded (400,000 cells/well) to 6-well plates. Experimental group consisted of duplicates of untreated control cells and cells treated with chaetocin (50 nM) for 24 h. RNA extraction was performed by Qiagen RNAeasy Mini Kit. Samples were sent to Berkeley University Functional Genomics Laboratory (Berkeley, CA) for sequencing at Illumina Hiseq4000 system to generate 50 bp single-end reads. Detailed methods on library preparation is described in Supplementary Info. Data have been deposited in NCBI’s Gene Expression Omnibus, accessible with GEO# GSE126462. Differentially expressed genes were identified based on negative binomial distribution using DESeq2(v.1.18.1)^33^. Enrichment of gene sets were analyzed using Gene Set Enrichment Analysis (GSEA) software^34^ to obtain enriched hallmark pathways related to drug treatment.

### In vitro ROS detection

U87MG cells were seeded (300.000 cells/well) in 6-well plates. NAC was applied 24 h prior to and during chaetocin treatment. Chaetocin treatment is started 3 h before the induction with ROS detection reagent and endured during the loading process, whereas pyocyanin is added right at the induction step. Cells were detached by trypsinization, collected, washed with wash buffer and centrifuged at RT. Cells were induced by loading with ROS/Superoxide detection mix (Abcam, ab139476 kit) supplemented with above mentioned treatments and incubated for 30 min in the cell culture incubator (37 °C, 5% CO_2_). Samples were kept on ice and analyzed with Flow Cytometry (BD Biosciences, USA) at FL1-A (green oxidative stress detection reagent) and FL2-A (orange, superoxide detection reagent) channels for 10.000 cells. Compensation correction was made to avoid overlap between green and orange fluorescent signals.

### H2AX staining

U87MG cells were seeded (25,000 cells/well) on glass coverslips in 24-well plates. Upon completion of treatment (simultaneous combinatorial treatment with Chaetocin and TRAIL), wells were washed with PBS (3 times) and cells were fixed using 100% ice-cold methanol. Cells were washed with PBS (3 times) and then incubated in blocking solution (5 ml Triton-X, 7,5 ml FBS, 37.5 ml PBS) for 15 min at RT. Cells were washed with PBS (3 times) and then incubated in primary antibody (Anti-phospho-Histone H2AX Ser139 Antibody, Millipore, 05–636, 1:100 diluted) at RT for 2 h (or overnight at 4 °C). Cells were washed with PBS (3 times) and incubated with secondary antibody (anti-Mouse IgG, Texas Red IR conjugated, 1:100 diluted) for 1 h at RT. After washing, coverslips were mounted in DAPI on microscope slides and visualized with Leica DMi8 inverted microscope (Leica Microsystems, Germany).

### Cloning

gRNA**:** In order to deplete the expression of selected genes such as DR5, Casp8, Bid, Casp3, Casp7, Suv39H1, and HMOX1 with CRISPR/Cas9 method, gRNAs were either derived from Gecko v2 library^35^ or designed against functional domains of genes using CCtop tool^36^. All gRNA sequences are presented in Supplementary Table 3. For cloning, top and bottom strands of gRNA against target genes were annealed and used for ligation into pLenti-CRISPR-V2 vector (for HMOX1), pLenti-CRISPR-V1 vector (for SUV39H1) and pLenti-Guide vector (for DR5, Bid, Casp8, Casp3, and Casp7), which was digested by BsmB1 followed by antarctic phosphatase treatment. Ligation was performed at RT for 15 min using Quick Ligase Kit (Roche, Switzerland) and ligation product was heat shock transformed to competent bacteria Stbl3. After growing and selecting in LB, plasmid isolation and diagnostic digestion was performed by MN miniprep kit, and plasmids were sent for sequencing. The efficiency of gRNAs was then verified in cells transduced with each vector using T7 Endonuclease Assay, as described in Supplemental Information. Efficient knockout with gRNA occurs within approximately 18 days since Cas9 activity takes time.

shRNA**:** shRNA sequences targeting Bcl2 and BclXL were designed using RNAi Codex program^37^. All oligo sequences are listed in Supplementary Table 4. These oligos were PCR-amplified by using following primers having compatible restriction ends with backbone vector, pSMP. F: 5′GATGGCTGCTCGAGAAGGTATATTGCTGTTGACAGTGAGCG-3′, R: 5′-CCCTTGAACCTCCTCGTTCGACC-3′. PCR products were cloned into pSMP retro-viral backbone as described^38^. All vectors were verified by sequencing.

Tet-TRAIL vector**:** DNA sequence producing secreted TRAIL protein was amplified from LV-TRAIL plasmid via primers containing BamHI and XbaI cut sites and ligated into pENTR1A plasmid (Addgene plasmid #17398). Then, Gateway cloning was performed to take pLIX_402 Tet-inducible lentiviral expression vector (Addgene plasmid #41394). Vectors were verified by sequencing. The efficiency of the Tet-TRAIL was tested in vitro after transduction of U87MG cells with lentiviruses and treating with different concentrations of Doxycycline (D9891 Sigma-Aldrich, MO, USA).

### Viral packaging and transduction

All lentiviral or retroviral vectors used to transduce GBM cells throughout this study are listed in (Supplementary Table 6) and all viral packaging was performed as described^38,39^. Briefly, on day0, 2.5 × 10^6^ 293T cells were seeded to 10 cm culture dish with DMEM supplemented with 10% FBS and 1% Pen/Strep. Viral plasmid DNA (2,5 µg) and packaging plasmids 8.2Dvpr and VSVG (2,5 µg) were transfected to cells using FugeneHD (Promega, USA). Next day, media of plate was changed and 48 and 72 h post transfection, media containing virus was collected. Viral media was aliquoted and stored at −80 °C. Cells were seeded at desired density and were transduced with virus containing media supplemented with protamine sulfate (10 µg/ml). Sixteen hours post-transduction, viral medium was replaced by fresh media. Transduced cells were selected by Puromycin at a final concentration of 1 μg/ml for 3 days. For constructs in pLenti-Guide backbone, cells were transduced with lentiCas9-Blast vector and selected with Blasticidin for 6 days prior to transduction with gRNAs.

### Patient survival analysis

Gene expression profiles of “glioblastoma multiforme” (GBM) and “brain lower grade glioma” (LGG) tumors were preprocessed by the unified RNA-Seq pipeline of the Cancer Genome Atlas (TCGA) consortium (https://portal.gdc.cancer.gov). For both cancer types, HTSeq-FPKM files of all primary tumors from the most recent data freeze (i.e., Data Release 14–December 18, 2018) were downloaded, leading to 703 files in total. Metastatic tumors were not included since their underlying biology would be very different than primary tumors. Clinical annotation files of cancer patients were used to extract their survival characteristics (i.e., days to last follow-up for alive patients and days to death for dead patients). For both cancer types, Clinical Supplement files of all patients from the most recent data freeze were downloaded, leading to 1114 files in total. To perform survival analysis using gene expression profiles, only patients with available survival information and gene expression profile were included, which led to a collection of 663 patients in total. The gene expression profiles of primary tumors were first log_2_-transformed and then *z*-normalized within each cohort before further analysis. The heat maps of gene expression values were based on these *z*-normalized gene expression values. For gene set analyses, 663 samples were grouped into two categories using *k*-means clustering (*k* = 2) on the *z*-normalized gene expression values of all genes included. Kaplan-Meier survival curves of these two groups were then compared. For single gene analyses, 663 samples were first grouped into two categories (i.e., low and high) by comparing each sample’s gene expression value against the mean expression value of that particular gene. Kaplan-Meier survival curves of these two groups were then compared. *p*-value obtained from the log-rank test performed on these two survival curves were displayed.

### In vivo experiment

Non-obese diabetic/severe combined immunodeficiency (NOD/SCID) mice housed and cared in appropriate conditions of Koç University Animal Facility were used and all protocols were approved by the institution boards of Koç University. Firefly Luciferase (Fluc) and mCherry expressing stable U87MG cells were generated by viral transduction as described^39^. mCherry expression was validated by fluorescence microscopy and Fluc activity was validated by utilizing in vitro luminescence assay and Synergy Biotek Plate reader. Before implantation, Fluc-mCherry expressing U87MG cells were transduced with Tet-TRAIL lentiviruses. For subcutaneous tumor implantation, 2 × 10^6^ were injected in 100 µl PBS per mouse (*n* = 5/group) into the flanks of mice. For orthotopic model, 1 × 10^5^ cells were injected in 7 µl PBS intracranially using stereotaxic injection, as described^40^. Tumor development was monitored by repeated noninvasive bioluminescence imaging (IVIS Lumina III) using 150 µg/g of D-Luciferin intraperitoneally. To test the effect of TRAIL and/or chaetocin, mice with established tumors were categorized into four experimental groups and doxycycline (10 mg/ml) and chaetocin (20 mg/kg) treatments were performed simultaneously as intraperitoneal injections (twice/week). Two weeks after treatment, mice were sacrificed, and tumors were dissected. Quantification of tumor progression was performed with GraphPad PRISM software (San Diego, CA, USA)

## Results

### Epigenetic compound screen identifies chaetocin as a novel TRAIL sensitizer

**Fig. 1:**
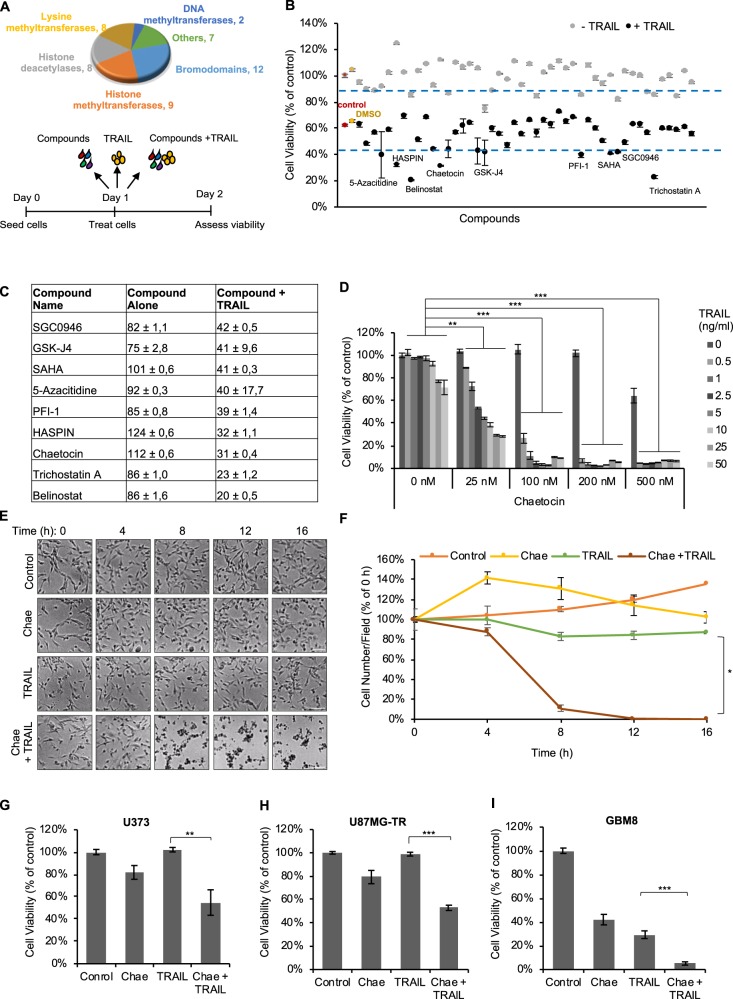
Epigenetic compound screen identifies chaetocin as TRAIL sensitizer. **a** Top: Chemical library composition of inhibitors of chromatin modifier proteins (12 Bromodomain inhibitors, 8 HDAC inhibitors, 9 HMT inhibitors, 8 HDM inhibitors, 2 DNMT inhibitors, 2 kinase inhibitors and 1 HAT inhibitor). *Bottom:* Schematic diagram of the experimental setup. **b** Plot of percent cell viability after treatment. Data were normalized to untreated control cells. Dotted lines denote 1 S.D. from % Mean cell viability upon treatment. Compounds lying below the lower threshold are TRAIL sensitizers. **c** List of compounds that augmented TRAIL response. **d** Viability analyses of U87MG cells showing markedly reduced viability upon Chaetocin and TRAIL combinational treatment at various dosages for 24 h. Data were normalized to untreated control. **e** Representative snapshot images from live cell imaging of U87MG cells upon chaetocin (100 nM) and TRAIL (100 ng/ml) combinatorial treatment for 16 h. Scale bar: 100 µm. **f** Quantification of live cell imaging data by ImageJ program through counting live/death cell percentage at each time point. **g** Viability analyses of innately TRAIL resistant U373 cells, **h** U87MG-TR cells with acquired TRAIL resistance and **i** primary GBM cell line GBM8 upon chaetocin and TRAIL combinatorial treatment chaetocin (100 nM) and TRAIL (100 ng/ml) for 24 h. Data were normalized to untreated control cells ((*), (**), and (***) denote *P* < 0.05, *P* < 0.01 and *P* < 0.001, respectively, two-tailed Student’s *t*-test).

To identify compounds that can sensitize GBM cells to TRAIL, we conducted a chemical screen in U87MG cells using a library composed of compounds targeting different classes of chromatin modifiers^31^. The screen assessed the effects of the inhibitors alone or in combination with a fixed concentration of TRAIL through ATP based cell viability assays (Fig. 1a). DMSO-only treated and untreated cells served as negative controls. On average, compounds alone had minimal effect on cell viability (98.8 ± 9.9%). To determine which compounds to follow-up, we took into account total variability across all compounds and considered a compound a hit if it reduced cell viability 1 SD or lower (88.9% for compound alone and 42.1% for TRAIL combination). Accordingly, 9 compounds significantly decreased viability (namely; HDAC inhibitors Belinostat, CI-994 and TrichostatinA, HDM inhibitors GSK-J4, JIB-04 and Tranylcypromine; bromodomain inhibitor PFI-1, HMT inhibitor SGC0946 and methyl-lysine reader domain antagonist UNC1215) on their own. The response to TRAIL alone was 62 ± 0.8% for control and 65 ± 1% for DMSO groups (Fig. 1b). When combined with TRAIL, compounds that decreased viability below 42.1% were SGC0946, GSK-J4, SAHA, 5-Azacytidine, PFI-1, HASPIN, chaetocin, TrichostatinA and Belinostat (Fig. 1c). After validating the hits from the screen (Supplementary Fig. 1A), we focused on those that did not reveal toxicity on their own but augmented the TRAIL-response of GBM cells. Those compounds were chaetocin, HASPIN, and SAHA. While SAHA, a well-known HDAC inhibitor, has been previously reported to cooperate with TRAIL^41^ and the antitumor role of protein kinase HASPIN has been established^42,43^, chaetocin has not been studied in relation to TRAIL in GBM. Therefore, we chose to further assess the effects of chaetocin, a fungal metabolite produced by *Chaetomium* fungal species that has antimicrobial and cytostatic activity^44^. Chaetocin is an unspecific inhibitor of lysine-specific histone methyltransferases including SU(VAR)3-9^45^ and also inhibits the oxidative stress mitigation enzyme thioredoxin reductase-1 (TrxR1 or TXNRD1)^46^. To assess the potential of chaetocin as a TRAIL sensitizer, we performed viability assays. Accordingly, Chaetocin combination sensitized U87MG cells to TRAIL in a dose-dependent manner, even at low doses which did not exert toxicity alone (Fig. 1d). Using CompuSyn software based on Chou-Talalay model for synergy quantification, we calculated combination index (CI) value for Chaetocin and TRAIL (Supplementary Fig. 1B). At effect level (Fa) > 0.5; Chaetocin and TRAIL combination yielded CI value smaller than 1, indicating strong synergism between the two drugs (Supplementary Fig. 1C–D).

To visualize the timing and mode of cell death, we performed live cell imaging on GBM cells. Chaetocin and TRAIL, when applied as single agents were not potent death inducers, however when applied in combination, they induced cell death significantly (Fig. 1e, Supplementary videos 1–4). The observed death involved membrane blebbing, cell shrinkage and formation of apoptotic bodies, as characteristic changes observed during apoptosis. Quantification of the number of cells that remain viable in response to treatment revealed significant cooperation of chaetocin and TRAIL in reducing cell viability (Fig. 1f). As U87MG exhibit only medium sensitivity to TRAIL^32^, we examined the effects of chaetocin in fully TRAIL-resistant cells lines as well. Using a resistant derivative of U87MG, U87MG-TR (manuscript under review) and innately resistant U373 cells, we showed that chaetocin could also sensitize these cells to TRAIL (Fig. 1g, h). This effect was observed in a sensitive primary GBM cell line GBM8 as well (Fig. 1i). Together, these findings suggest that chaetocin is a potent agent to overcome TRAIL resistance and augment TRAIL response of GBM cells.

### Combined chaetocin and TRAIL treatment leads to efficient apoptosis of GBM cells

**Fig. 2:**
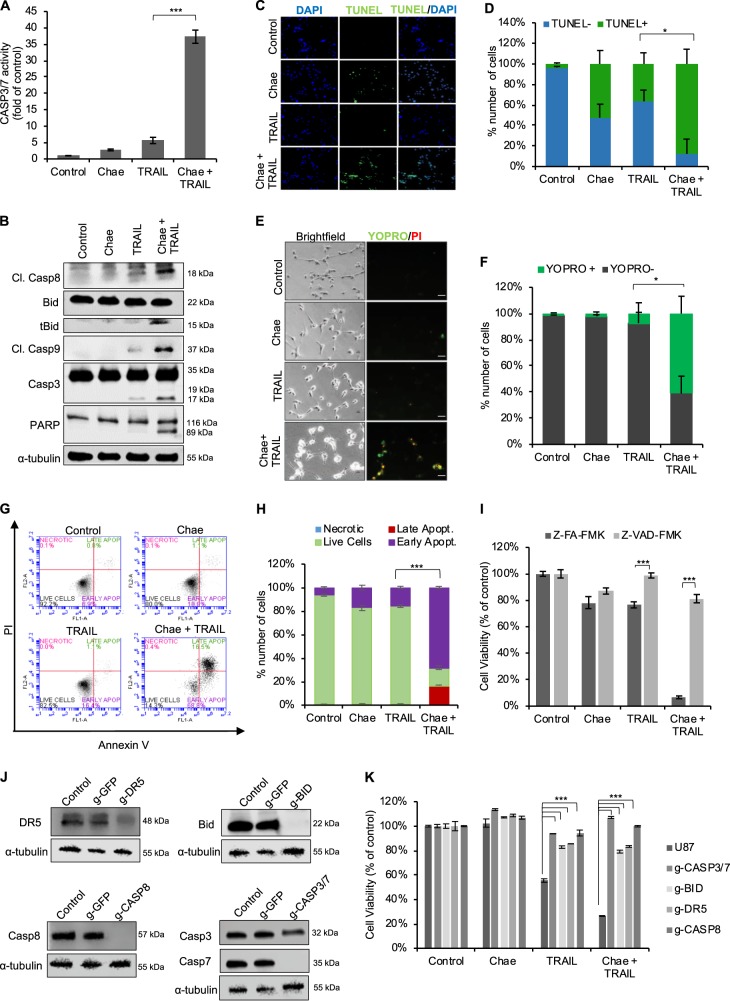
GBM cells display augmented apoptotic response upon chaetocin and TRAIL combinatorial treatment. **a** Caspase-3/7 activity analyses of U87MG cells upon chaetocin (100 nM) and TRAIL (100 ng/ml) combinatorial treatment. Data were normalized to untreated control cells. **b** Western blot analyses of U87MG cells for cleaved Casp8, Bid, t-Bid, Casp3 and PARP after pretreatment with chaetocin (100 nM for 24 h) followed by 6 h TRAIL (100 ng/ml) treatment. α**-**tubulin was shown as protein loading control. **c** Terminal deoxynucleotidyl transferase dUTP nick end labeling (TUNEL) assay on U87MG cells showing increased DNA fragmentation upon chaetocin (100 nM) and TRAIL (100 ng/ml) combinatorial treatment for 24 h. Blue: DAPI staining nuclei, Green: TUNEL (+) cells. Scale bar: 100 µm. **d** Quantification of TUNEL staining by ImageJ program through counting TUNEL (+) cells with green fluorescence. **e** YO-PRO-1 and PI staining upon chaetocin (100 nM) and TRAIL (100 ng/ml) combinatorial treatment of U87MG cells for 24 h. Green: YO-PRO-1 staining apoptotic cells, Red: PI staining dead/necrotic cells. Scale bar: 200 µm. **f** Quantification of YO-PRO-1/PI staining by ImageJ program through counting green and red fluorescence positive cells. **g** Flow cytometric analysis of Annexin V/PI stained U87MG cells upon chaetocin (100 nM) and TRAIL (100 ng/ml) combinatorial treatment for 24 h. **h** Quantification of flow cytometry data showing marked increase in apoptotic cell populations upon combinatorial treatment. Data were normalized to total number of cells under each condition. **i** Cell viability analysis U87MG cells pretreated with caspase inhibitors (20 µM for 24 h) followed by chaetocin (100 nM) and TRAIL (100 ng/ml) treatment for 24 h in presence of inhibitors. Z-FA-FMK: negative control, Z-VAD-FMK: general caspase inhibitor. **j** Western blot analyses of U87MG cells showing individual stable CRISPR knockouts of DR5, BID, and Casp8, as well as double knock out Casp3/7 genes. GFP targeting guide RNA (g-GFP) was used as negative control for CRISPR assay. α-tubulin was shown as protein loading control. **k** Viability analysis of CRISPR edited U87MG cells upon combinatorial treatment with chaetocin (100 nM) and TRAIL (100 ng/ml) for 24 h. Data were normalized to untreated control. ((*) and (***) denote *P* < 0.05 and *P* < 0.001, respectively, two-tailed Student’s *t*-test).

To address whether the observed death upon combinatorial treatment indeed involves apoptosis, we investigated caspase activity of GBM cells. While chaetocin or TRAIL as single agents only moderately increased Casp3/7 activity, combinatorial treatment resulted in major elevation of Casp3/7 activity (Fig. 2a, Supplementary Fig. 2A–B). Similarly, enhanced cleavage of both initiator (Casp8) and effector (Casp9, Casp3) caspases were evident in combinatorial treatment, as revealed by western blotting. Furthermore, significant truncation and activation of Bid, a link between extrinsic and intrinsic apoptosis, was detected when GBM cells were treated with both chaetocin and TRAIL. Finally, as an important hallmark of apoptosis, cleavage of Poly (ADP-ribose) polymerase-1 (PARP) was also markedly enhanced upon combinatorial treatment (Fig. 2b).

To examine the apoptotic features of GBM cells, we performed TUNEL assay, which detects fragmented DNA generated during apoptosis^47^ and showed TUNEL-positive cells were significantly more abundant in GBM cells sensitized to TRAIL through chaetocin (Fig. 2c, d). Similarly, in a fluorescence dye-based “live/dead assay”, we observed significant increases in the percentage of apoptotic cells upon combinatorial treatment (Fig. 2e, f). These results were supported by flow cytometric analysis of AnnexinV-positive and PI-positive cells, where the presence of enhanced early and late apoptotic cells were evident with both chaetocin and TRAIL treatment (Fig. 2g, h, Supplementary Fig. 2C–D) indicating that chaetocin and TRAIL cooperate to induce apoptosis in GBM cells.

To further validate these findings, we investigated the functional effect of caspase activation using general caspase inhibitor Z-VAD-FMK. Accordingly, the inhibitor interfered with TRAIL sensitizing effect of chaetocin and markedly reduced cell death (Fig. 2i, Supplementary Fig. 2E). Next, we generated CRISPR-Cas9 mediated ablation of apoptosis-mediator proteins DR5, Casp8, Bid, Casp7, and Casp3 in U87MG cells (Fig. 2j). While individual knockout of the major components of extrinsic apoptosis pathway, DR5 and Casp8, recovered the cell death induced by chaetocin and TRAIL, the knockout of either Casp3 or Casp7 alone were not sufficient to recover the chaetocin induced TRAIL sensitization (data not shown). When both effector caspases were simultaneously ablated, there was a recovery in the response of GBM cells. Similarly, the reduction of Bid levels led to effective recovery of cell viability upon combinatorial treatment (Fig. 2k). Taken together, our data demonstrate that chaetocin-induced TRAIL sensitization involves the activation of major apoptotic machinery in GBM cells.

### Chaetocin effectively sensitizes GBM cells to other pro-apoptotic agents, such as FasL and BH3 mimetics

**Fig. 3:**
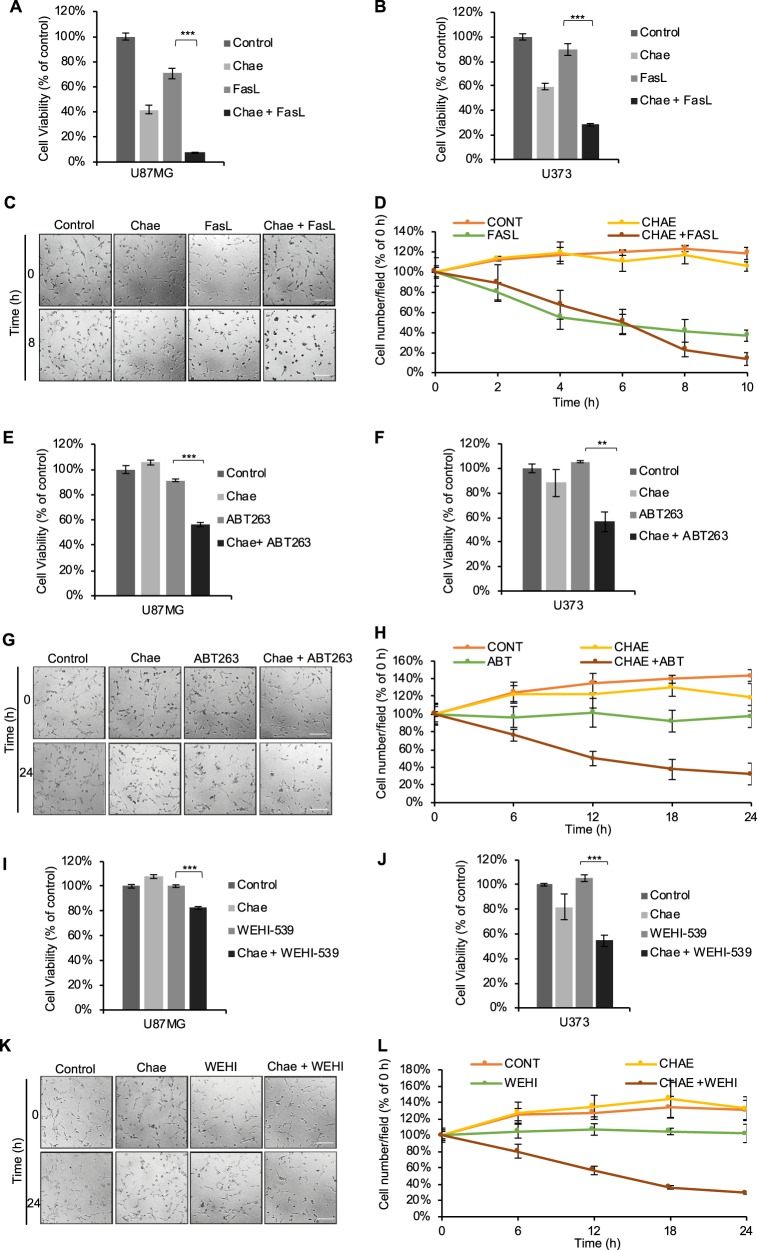
Chaetocin effectively sensitizes GBM cells to other pro-apoptotic agents, FasL and BH3 mimetics. **a**, **b** Viability analyses of U87MG (**a**) and U373 (**b**) cells showing markedly reduced viability upon combinatorial treatment with chaetocin (100 nM) and FasL (100 ng/ml) for 24 h. Data was normalized to untreated control. **c** Representative snapshot images from live cell imaging of U87MG cells upon chaetocin (100 nM) and FasL (200 ng/ml) combinatorial treatment for 10 h. Scale bar: 200 µm. **d** Quantification of live cell imaging by ImageJ program through counting live/dead cell percentage at each time point. **e**, **f** Viability analyses of U87MG (**e**) and U373 (**f**) cells showing significantly reduced viability upon combinatorial treatment with chaetocin (100 nM) and ABT263 (1 µM) for 24 h. **g** Representative snapshot images from live cell imaging of U87MG cells upon chaetocin (100 nM) and ABT263 (1 µM) combinatorial treatment for 24 h. Scale bar: 200 µm. **h** Quantification of live cell imaging by ImageJ program through counting live/dead cell percentage at each time point. **i**, **j** Viability analyses of U87MG (**i**) and U373 (**j**) cells showing significantly reduced viability upon combinatorial treatment with chaetocin (100 nM) and WEHI-539 (1 µM) for 24 h. **k** Representative snapshot images from live cell imaging of U87MG cells upon chaetocin (100 nM) and WEHI-539 (1 µM) combinatorial treatment for 24 h. Scale bar: 200 µm **l** Quantification of live cell imaging by ImageJ program through counting live/dead cell percentage at each time point. ((**) and (***) denote *P* < 0.01 and *P* < 0.001, respectively, two-tailed Student’s *t*-test).

To evaluate whether chaetocin mediated apoptotic sensitization is exclusive to TRAIL or whether it can be a general sensitizer for apoptosis, we explored the effect of chaetocin in combination with further pro-apoptotic agents. Chaetocin effectively sensitized GBM cells, as well as U87MG-TR cells (Supplementary Fig. 3A) to FasL, another extrinsic apoptosis ligand (Fig. 3a–d, Supplementary Videos 5–8), as revealed by end-point cell viability assays (Fig. 3a, b) and live cell imaging (Fig. 3c, d). In addition, depletion of Casp8, but not DR5, recovered chaetocin mediated sensitization to FasL (Supplementary Fig. 3B). The effect of chaetocin was also tested in combination with BH3 mimetics ABT263 (Bcl2 and BclXL dual inhibitor^48^) and WEHI539 (BclXL inhibitor^49^), which are intrinsic apoptosis inducers. Chaetocin was found to be a strong sensitizer against these intrinsic apoptosis inducers (Fig. 3e–h, i–l and Supplementary Fig. 4A–B, Supplementary Videos 9–12 and 13–16). Taken together, these results show that chaetocin cooperated with several apoptotic agents to induce apoptosis in GBM cells.

### Manipulation of the intrinsic apoptosis machinery regulates the chaetocin-mediated TRAIL sensitization

**Fig. 4:**
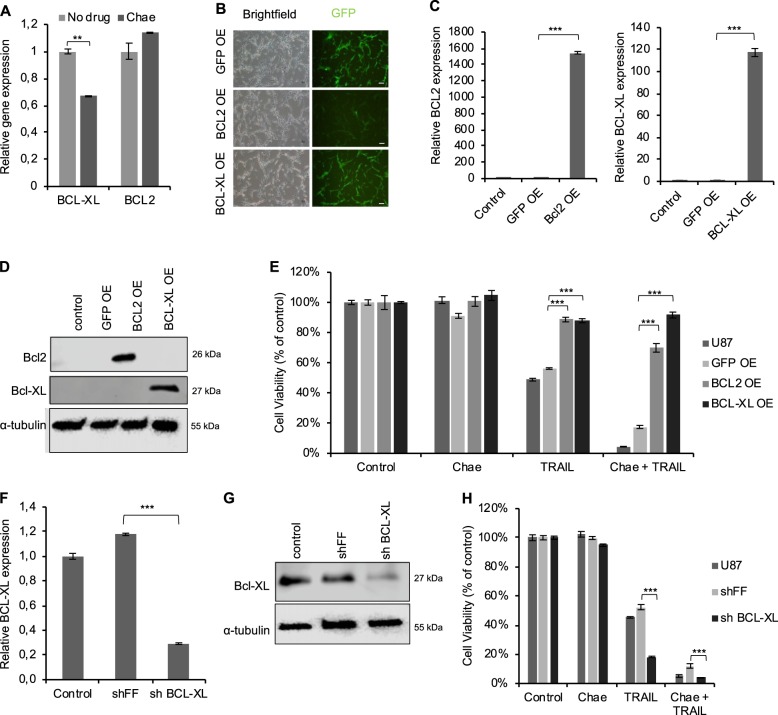
Manipulation of the intrinsic apoptosis machinery regulates chaetocin-mediated TRAIL sensitization. **a** qPCR analysis showing Bcl2 and BclXL mRNA levels in U87MG cells upon chaetocin treatment (100 nm, 24 h). Data were normalized to no drug conditions. **b** Representative images showing GFP signal of U87MG cells transduced with GFP, Bcl2 and BclXL overexpression constructs with an incorporated GFP. Scale bar: 200 µm. **c** qPCR analysis confirming elevated Bcl2 and BclXL mRNA levels in transduced U87MG cells. **d** Western Blot showing overexpression of Bcl2 and BclXL proteins. α**-**tubulin was shown as protein equal loading control. **e** Viability analysis of U87MG cells overexpressing either Bcl2 and BclXL proteins or negative control GFP upon chaetocin (100 nM) and TRAIL (100 ng/ml) combinatorial treatment. **f** qPCR illustrating shRNA mediated knockdown of BclXL protein in U87MG cells. shFF is negative control shRNA. **g** Western Blot showing the knockdown of BclXL protein levels. α**-**tubulin was shown as protein loading control. **h** Cell viability assay of U87MG cells showing further sensitization of cells to chaetocin + TRAIL mediated apoptosis upon BclXL knockdown. ((**) and (***) denote *P* < 0.01 and *P* < 0.001, respectively, two-tailed Student’s *t*-test).

Intrinsic apoptosis ultimately leads to reduction of mitochondrial integrity^50^, where release of Cytochrome C is regulated by the expression and activity of anti-apoptotic Bcl2 and BclXL proteins. Following up on our findings that chaetocin cooperates with Bcl2/BclXL inhibitors (Fig. 3e–l), we further examined whether genetic manipulation of Bcl2 and/or BclXL could change the chaetocin-mediated TRAIL sensitization in GBM cells. Endogenous expression of BclXL, but not Bcl2 levels were significantly affected by chaetocin treatment (Fig. 4a). In a gain-of-function approach, we overexpressed Bcl2 or BclXL using retroviral vectors that co-expressed GFP (or GFP alone as controls) (Fig. 4b–d). Bcl2 or BclXL expression rendered U87MG cells more resistant to apoptosis induced both by TRAIL-only or combinatorial treatment (Fig. 4e). Conversely, in a loss-of-function approach using shRNA vectors, BclXL expression was efficiently downregulated at the mRNA and protein level (Fig. 4f–g) which led to further augmentation of TRAIL sensitization in U87MG cells (Fig. 4h), as well as reducing TRAIL resistance in the fully resistant U373 cells (Supplementary Fig. 5). Taken together, these results show that Bcl2/BclXL play critical roles in chaetocin-mediated TRAIL sensitization in GBM cells which emphasized the active role of mitochondria in sensitization process.

### Chaetocin-induced global transcriptome changes reveal the alteration of important hallmarks of cancer

**Fig. 5:**
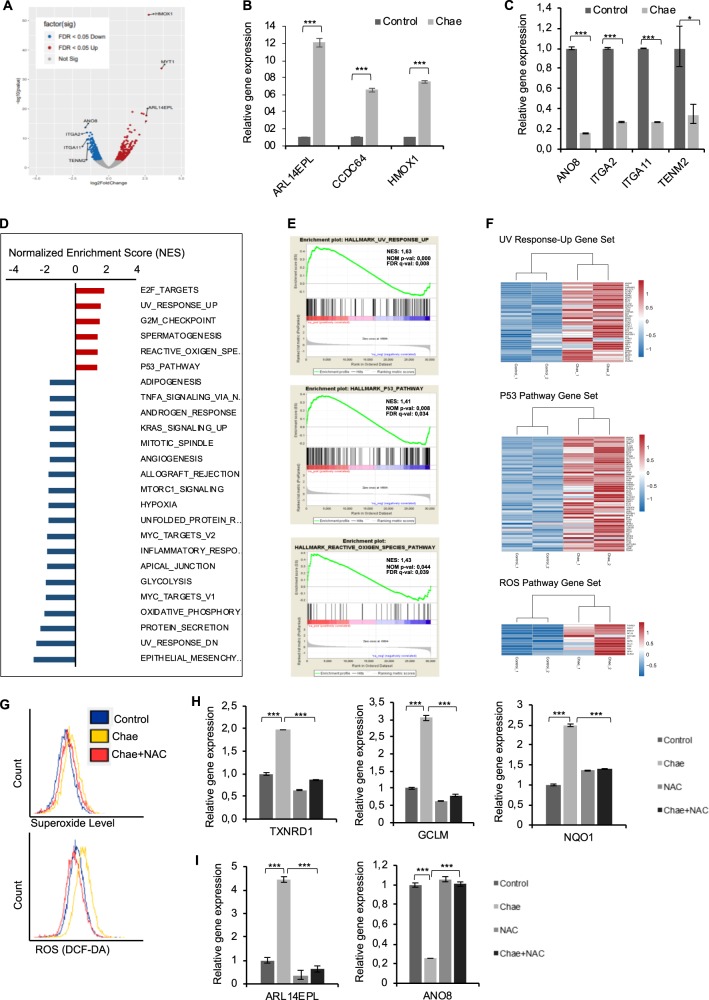
Chaetocin-induced global transcriptome changes reveal the alteration of important hallmarks of cancer. **a** Volcano plot of RNA sequencing data showing significantly (*p* < 0.05) up and down regulated genes by chaetocin (50 nM, 24 h) based on their log2 transformed expression data with false discovery rate (FDR) threshold of 0.05. **b**–**c** qPCR validation of top 4 upregulated (*MLC1, ARL14EPL, HMOX1, CCDC64*) (**b**) and downregulated (*ANO8, ITGA2, ITGA11, TENM2)* (**c**) genes obtained from RNAseq analysis. Data were normalized to untreated control. **d** Graph represents Gene Set Enrichment Analysis (GSEA) results pointing out chaetocin mediated positively and negatively enriched hallmark pathways based on their Normalized Enrichment Score (NES). **e** Representative enrichment plots for hallmark UV response-up, p53 and reactive oxygen species pathways. **f** Heatmaps of genes listed under UV response-up, p53 and ROS pathways from GSEA revealing significantly upregulated genes upon chaetocin treatment. Expression data were normalized to control condition and log 2 transformed (*p* < 0.05). **g** DCFDA flow cytometric analysis of ROS generation in U87MG cells treated with chaetocin in the presence or absence of N-acetyl-cysteine (NAC). **h** qPCR analysis showing that chaetocin treatment (100 nm, 24 h) upregulated *TXNRD1, GCLM*, and *NQO1* gene levels in ROS-dependent manner. Data were normalized to no drug conditions. **i** qPCR analysis showing modulation of *ARL14EPL* and *ANO8* gene levels by chaetocin treatment (100 nm, 24 h) in ROS-dependent manner. Data were normalized to Control conditions. ((*) and (***) denote *P* < 0.05 and *P* < 0.001, respectively, two-tailed Student’s *t*-test).

Chaetocins’ potency as general apoptosis-sensitizer prompted us to check its effect on apoptosis related gene expression. In U87MG cells, chaetocin positively modulated the expression of pro-apoptotic genes such as *PUMA, NOXA, HRK, BIM, BAD, DR4, CASP3*, and *CASP7*, whereas downregulated anti-apoptotic genes such as *CIAP1, CIAP2*, and *MCL1* (Supplementary Fig. 6A). We then performed global transcriptional profiling using RNA sequencing (RNAseq) to analyze the chaetocin-mediated changes at the whole transcriptome. A volcano plot for fold-changes in gene expression illustrated that 373 genes were up-regulated and 478 genes were down-regulated significantly (FDR < 0.05) upon 24 h treatment with a low dose (50 nM) chaetocin (Fig. 5a). Changes in the expression of top scoring genes (*HMOX1, MLC1, ARL14EPL, ANO8, ITGA2, ITGA11*, and *TENM2*) were validated by qPCR (Fig. 5b, c).

We performed Gene Set Enrichment Analysis (GSEA)^34^ and observed that E2F targets, UV response up, G2M checkpoint, ROS and p53 pathways were among top positively regulated, and EMT, UV-response down, protein secretion and oxidative phosphorylation pathways were among top negatively regulated hallmark pathways (Fig. 5d, e, Supplementary Fig. 7). The heatmaps of genes positively contributing to each enrichment plot revealed significant differences in the expression patterns of UV-response up, p53 and ROS pathway (Fig. 5f). To validate the implications from the GSEA data, we tested the effect of chaetocin on cell cycle distribution (as an output for G2-M checkpoint) and cellular invasion (as output for EMT deregulation) (Supplementary Fig. 8A–B). PI staining revealed cell cycle arrest induction with a significant decrease in the S phase and an increase in G2/M phase following chaetocin treatment in U87MG and U373 cells. Spheroid invasion assay to measure the invasive ability of GBM cells showed a reduction of dispersal upon chaetocin treatment, supporting the negative effect of chaetocin on EMT. ROS was higher upon chaetocin treatment, supporting the earlier findings on chaetocin mediated ROS induction^51^. As expected, the ROS scavenger N-acetyl-L-cysteine (NAC) reduced the level of ROS generated by chaetocin (Fig. 5g). Further evidence for chaetocin-mediated induction of the ROS pathway and its role during apoptosis is elevated expression levels of antioxidant genes such as *TXNRD1, GCLM*, and *NQO1* (Fig. 5h) and pro-apoptotic mediators such as *FADD, CASP3*, and *BIM*, which could be blocked with NAC treatment (Supplementary Fig. 6B). Chaetocin-induced changes in expression levels of other genes such as *ARL14EPL* and *ANO8* were also ROS-dependent (Fig. 5i, Supplementary Fig. 9A, B).

### Chaetocin mediated apoptosis sensitization of GBM cells is through ROS generation and consequent DNA damage induction

**Fig. 6:**
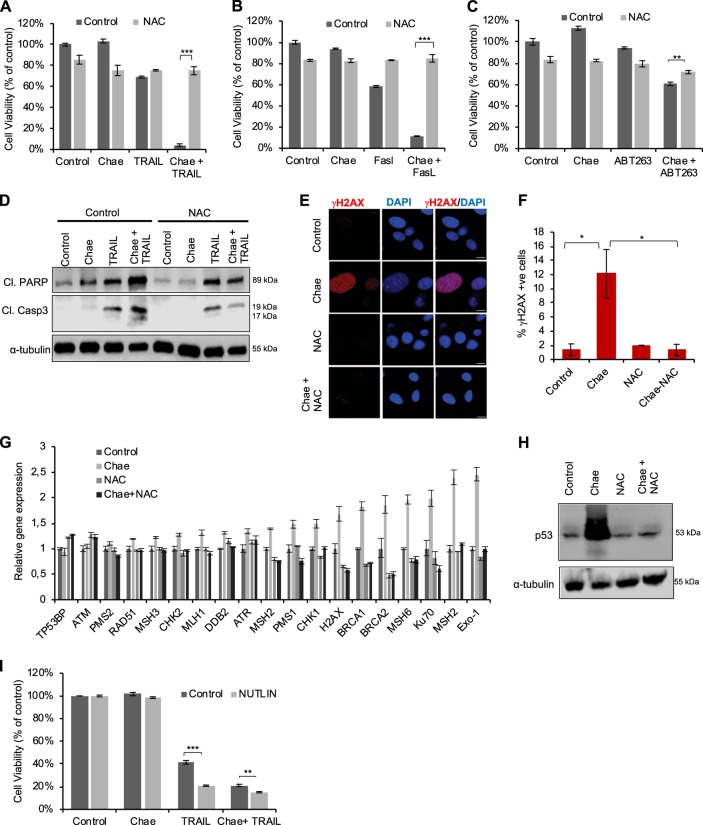
Chaetocin mediated apoptosis sensitization of GBM cells is through ROS generation and consequent DNA damage induction. **a** Viability analysis of chaetocin and TRAIL treated cells (100 nM, 100 ng/ml, respectively for 24 h) in presence and absence of NAC (10 µM). **b** Viability analysis of chaetocin and FasL treated cells (100 nM, 100 ng/ml, respectively for 24 h) in presence and absence of NAC (10 µM). **c** Viability analysis of chaetocin and ABT263 treated cells (100 nM, 1 µM, respectively for 24 h) in the presence or absence of NAC (10 µM). **d** Western blot showing the effect of NAC (10 µM) on activation of main players of apoptosis by chaetocin + TRAIL combinatorial treatment. **e** Representative fluorescent images from Phospho-H2AX (Ser139) staining showing DNA damage by prolonged exposure to chaetocin (100 nM, 24 h), which was blocked by pretreatment with NAC (10uM). Red: H2AX, Blue:DAPI. Scale bar: 10 µm. **f** Quantification of Phospho-H2AX (Ser139) staining. Number of % positive cells was plotted by counting the cells having more than 5 foci. **g** qPCR analysis revealing the modulation of DNA damage related gene expressions; specifically, those involved in mismatch repair pathway (*MSH2, MSH6, KU70*, and *EXO2*) and base excision repair (*BRCA1* and *BRCA2*) by chaetocin in ROS-dependent manner. NAC pretreatment (10 μM) was performed for 24 h prior to chaetocin (100 nM, 24 h). **h** Western blot analysis revealing accumulation of p53 protein in chaetocin (100 nM, 24 h) treated cells in ROS-dependent manner. α-tubulin was shown as loading control. **i** Viability analysis showing that pretreatment with p53 activator NUTLIN (10 mM, 24 h) increased the response of U87MG cells to chaetocin + TRAIL treatment (100 nM, 100 ng/ml, respectively). Data were normalized to untreated control. ((*), (**), and (***) denote *P* < 0.05, *P* < 0.01 and *P* < 0.001, respectively, two-tailed Student’s *t*-test).

To assess the role of ROS in chaetocin mediated apoptosis sensitization, we performed cell viability assays in the presence of NAC. Indeed, NAC treatment completely abolished chaetocin-mediated sensitization to TRAIL (Fig. 6a), to FasL (Fig. 6b) and to BH3 mimetic (Fig. 6c) in GBM and U87MG-TR cells (Supplementary Fig. 10), where PARP and Casp3 cleavage induced by chaetocin and TRAIL treatment was reduced in the presence of NAC (Fig. 6d).

Since ROS is general DNA damage inducer, we examined the role of chaetocin treatment on DNA damage. We analyzed canonical markers of DNA damage such as phospho-H2AX foci formation. Accordingly, ƔH2AX staining revealed increased DNA damage by prolonged exposure to chaetocin, which could be blocked by NAC treatment (Fig. 6e, f). We observed expression changes of DNA damage related genes; including mismatch repair (*MSH2, MSH6, KU70*, and *EXO2)* and base excision repair (*BRCA1* and *BRCA2*) (Fig. 6g).

As p53 activation was one of the top enriched gene sets from GSEA, we measured p53 protein levels and observed accumulation of p53 protein in chaetocin treated cells in a ROS-dependent manner (Fig. 6h). The relationship between chaetocin treatment, p53 and TRAIL sensitization was further evaluated using a p53 reporter system in HCT116 colon cancer cells (Supplementary Fig. 11A, B). Also, p53 knockout rendered HCT116 cells slightly resistant to chaetocin-mediated TRAIL sensitization (Supplementary Fig. 11C). NUTLIN, a well-studied MDM2 antagonist and p53 activator^52^ sensitized U87MG cells to TRAIL, providing further evidence for p53 in the chaetocin-induced apoptotic process (Fig. 6i). To rule out the changes in the senescence state due to high p53 activity upon chaetocin treatment, we performed X-gal staining revealing no changes in senescence (Supplementary Fig. 12).

### Heme Oxygenase 1 (HMOX1) regulates chaetocin-induced apoptotic sensitization

**Fig. 7:**
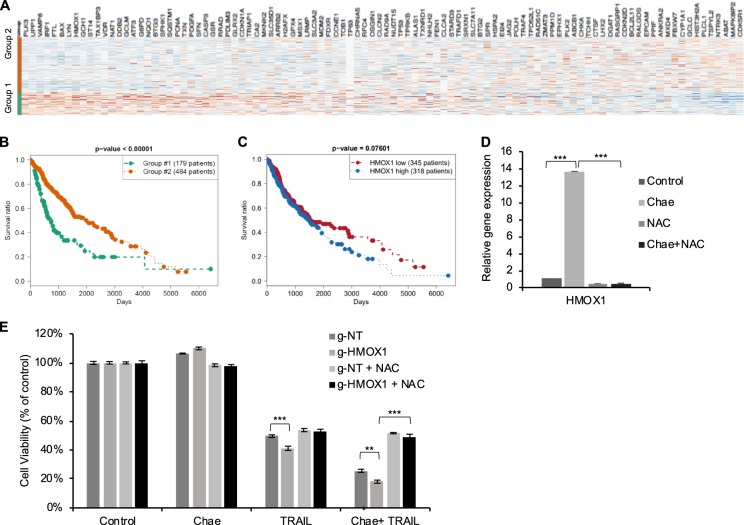
Depletion of HMOX1, a p53 driven and UV induced gene, further potentiates the response of GBM cells to chaetocin and TRAIL. **a** Glioma patients (*n* = 663, composed of LGG and GBM patients) from TCGA database were clustered within two distinct groups based on their expression of p53, UV response and ROS related genes from GSEA. **b** Survival curve for TCGA analysis on 2 distinct groups revealing significant survival difference. **c** Survival curve of glioma patients (from TCGA database) showing inverse correlation between patient survival and HMOX1 gene expression. **d** qPCR data showing upregulation of HMOX1 upon chaetocin treatment in ROS-dependent manner. Data were normalized to untreated control. **e** Viability analysis showing that HMOX1 knock out sensitized U87MG cells further to TRAIL and chaetocin + TRAIL in ROS-dependent manner. NAC pretreatment (10 μM) was performed for 24 h. Data were normalized to untreated control. ((**) and (***) denote *P* < 0.01 and *P* < 0.001, respectively, two-tailed Student’s *t*-test).

Enrichment of UV response, ROS, and p53 as hallmark pathways by GSEA led us to examine whether these chaetocin-induced changes can be recognized in the context of clinical information. We curated a list of genes that were significantly altered within the UV response, ROS, and p53 gene sets and correlated them with glioma patient survival using available TCGA data of GBM and lower grade glioma patient data. We first grouped a total of 663 patient samples into two categories using *k*-means clustering (*k* = 2) on the *z*-normalized gene expression values. Comparing the Kaplan-Meier survival curves of these two groups, we observed a significant clustering and survival difference between the groups (Fig. 7a, b). Heme oxygenase1 (HMOX1) was among the top most upregulated gene both by p53 and UV (Fig. 5f) and RNAseq data. HMOX1 is an essential enzyme for heme catabolism^53^. HMOX1 cleaves heme to form biliverdin and carbon monoxide, which exhibit anti-oxidant and anti-inflammatory functions, respectively^53^. Targeting HMOX1 was previously shown to be an effective approach to overcome therapy resistance of hormone-refractory prostate cancer^54^, urothelial and pancreatic cancers^55,56^. HMOX1 expression levels, when analyzed alone, was inversely correlated with glioma patient survival, although not highly significant (Fig. 7c). Since the expression of HMOX1 was significantly modulated by chaetocin (Fig. 7d) we asked whether HMOX1 could be within the regulatory axis during sensitization of GBM cells to TRAIL. We ablated HMOX1 in U87MG cells by CRISPR/Cas9 (Supplementary Fig. 13). Cells that lost HMOX1 expression were more sensitive to TRAIL and combinatorial treatments, again highlighting a critical role for ROS formation (Fig. 7e).

Chaetocin is an unspecific inhibitor of histone methyltransferases, including the H3K9 histone methyltransferase SUV39H1. In addition to its direct inhibition by chaetocin, SUV39H1 was previously shown to be indirectly modulated by cellular ROS produced by chaetocin^57^. We wondered whether the observed TRAIL sensitization is modulated through SUV39H1 inhibition. We generated SUV39H1 knockout U87MG cells (Supplementary Fig. 14A) and checked their TRAIL response. Depletion of SUV39H1 failed to sensitize cells further to apoptosis and rendered them slightly more resistant to TRAIL (Supplementary Fig. 14B). H3K9me(3) levels remained unchanged upon chaetocin treatment indicating that SUV39H1 inhibition is not the root cause for the pro-apoptotic effects of chaetocin (Supplementary Fig. 14C).

### Chaetocin and TRAIL treatments cooperate to reduce tumor growth in vivo

**Fig. 8:**
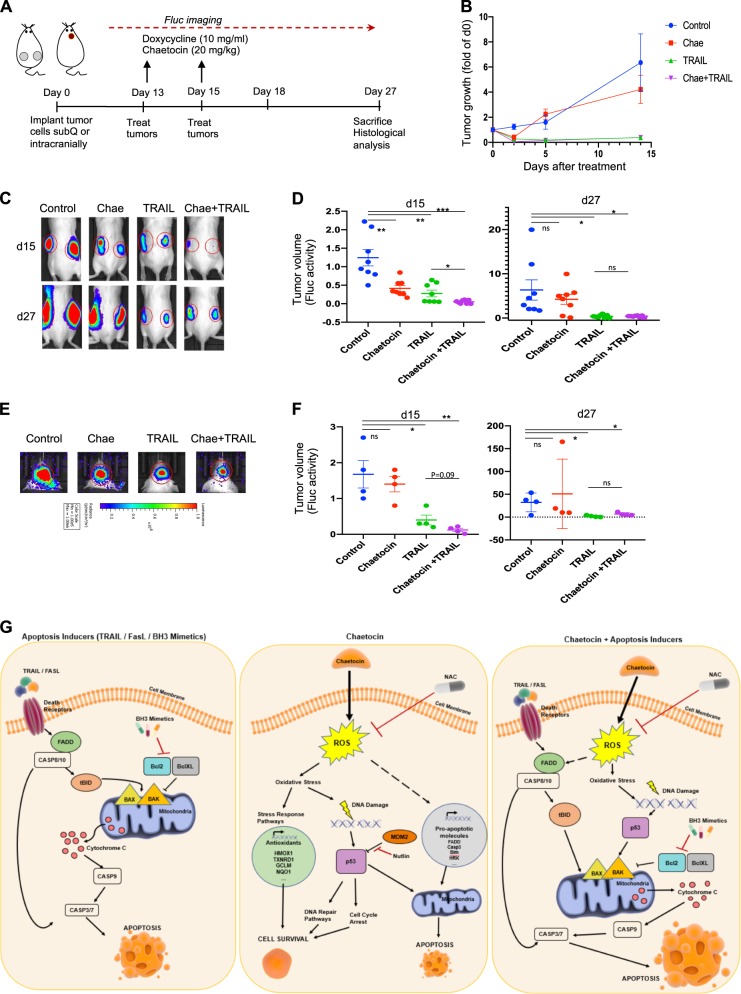
Chaetocin and TRAIL cooperate to reduce tumor growth in vivo. **a** Schematic description of the in vivo experiments. U87MG cells expressing Fluc and mCherry together with an inducible TRAIL vector (Tet-TRAIL) were injected subcutaneously or intracranially to non-obese diabetic/severe combined immunodeficiency (NOD/SCID) mice. Tumor cell injection was confirmed with noninvasive bioluminescence imaging (BLI) on day 0. After tumors are established, chaetocin + Dox administration was performed at Days 13 and 15. Tumor growth was monitored until Day 27 with BLI. **b** Graph depicting tumor growth as measured by bioluminescent radiance on 4 time points for 14 days (after treatment). Data were normalized to day 13 (day 0 of drug treatment) signal of each group (*n* = 8 tumors per group). **c** Representative images of bilateral tumors from days 15 and 27 displaying normalized bioluminescent efficiencies acquired (blue to red indicates lower to higher radiance as photons/s/cm2/steradian). **d** Plots depicting tumor volumes of each subQ tumor on d15 (left) and d27(right). **e** Representative images of intracranial tumors on d27. **f** Plots depicting tumor volumes of each intracranial tumor on d15 (left) and d27(right) (*n* = 4/group). ((*), (**), and (***) denote *P* < 0.05, *P* < 0.01and *P* < 0.001, respectively, unpaired parametric *t*-test) **g** Representative model illustrating chaetocin’s mode of action. Extrinsic apoptosis inducers such as TRAIL and FasL bind death receptors and lead to FADD mediated Casp8 activation. Active Casp8 cleaves and activates effector Casp3/7 and leads to apoptosis (extrinsic). Casp8 also truncates BID and cause BAX and BAK oligomerization in mitochondrial outer membrane that leads to Cytochrome C release, consequent activation of Casp9 and effector caspases3/7 and apoptosis (intrinsic). Intrinsic apoptosis can also be triggered by BH3 mimetics, which inhibit Bcl2 and BclXL antiapoptotic proteins thus facilitating BAX and BAK activity. Chaetocin elevates cellular ROS levels which cause DNA damage mediated p53 activation. Active p53 leads to cell cycle arrest and trigger DNA repair mechanism rendering mitochondria primed for apoptosis in case damage is unrepairable. ROS also elevates the expression of proapoptotic genes such as FADD and Casp3 and contributes to initiation of apoptosis. Antioxidant defense mechanisms get activated in response to chaetocin treatment to eradicate the detrimental effect of cellular ROS accumulation. When chaetocin is combined with apoptosis inducers, chaetocin mediated primed state of mitochondria, as well as elevated proapoptotic gene expression render cells much more prone to apoptosis induced by any extrinsic (TRAIL/FasL) or intrinsic (BH3 mimetics) stimuli.

To explore the efficacy of TRAIL sensitization by chaetocin in vivo, we examined subcutaneous (subQ) and orthotopic xenograft models of U87MG cells expressing firefly luciferase (Fluc) and mCherry (Fig. 8a). To supply tumors with TRAIL on-site, we developed tetracycline-inducible TRAIL vector, whose presence on its own was not toxic to U87MG cells. However, with doxycycline (Dox) treatment, TRAIL secretion was sufficient to markedly reduce U87MG cell viability and tumor growth (Supplementary Fig. 15A–C). In the subcutaneous model, Dox and chaetocin treatments were performed simultaneously and tumor growth was observed over 2 weeks (Fig. 8b). Chaetocin + TRAIL treatment attenuated subcutaneous tumor growth faster in comparison to the TRAIL only group, which was most notable right after treatment (d15); but overall effects of the TRAIL and combinatorial treatments were similar at d27 (Fig. 8c–e). Similarly, intracranial tumor volumes decreased more rapidly in combination treatment group at d15 though the effects became similar at day 27 (Fig. 8f, g). Representative subcutaneous tumors resected from sacrificed mice are illustrated (Supplementary Fig. 15D). Together, these results suggest that chaetocin and TRAIL combination might serve as efficient therapies for GBM models.

## Discussion

In this study, we interrogated the effects of epigenetic modifying compounds on GBM cell apoptosis in a screening approach and identified chaetocin as a novel sensitizer for apoptotic therapies in GBM cells. Our study explored the chaetocin-induced global effects and sensitizing ability in GBM cells. We showed that the effects of chaetocin on GBM cell apoptosis are unrelated to the alleged effect of chaetocin as SUV9H1 inhibitor; but are through generation of ROS and DNA damage induction leading to a p53 induced pro-apoptotic program. Furthermore, we demonstrated that chaetocin effectively cooperates with TRAIL, FasL, and BH3 mimetics ABT263 and WEHI539 to induce apoptosis in GBM cells. Finally, chaetocin and TRAIL combination treatment revealed efficacy in reducing tumor growth in vivo.

In the screen that involved chemical probes against chromatin modifiers, we identified HDAC inhibitors (Belinostat, Trichostatin A and SAHA) in accordance with their established role in apoptosis sensitization^58^. We also identified chaetocin as a novel apoptosis regulator in GBM cells. Whilst the relation of chaetocin with death receptor-dependent apoptosis was previously reported^59,60^ and the synergistic cytotoxicity of chaetocin with other epigenetic drugs such as SAHA, JQ-1^61^, Trichostain A^62^, Vorinostat, and AraC^57^ was previously explored in other cancers, no attempt was made to investigate effect of sub-toxic dose of chaetocin in combination therapies with pro-apoptotic agents. We here demonstrate that low dose chaetocin treatment is sufficient to induce cell death in combination with pro-apoptotic agents such as TRAIL and BH3 mimetics, suggesting that a brief treatment with chaetocin might be sufficient to prime GBM cells for apoptotic agents. The cooperation between chaetocin and TRAIL involved canonical apoptosis pathways, activation of effector caspases and regulation of DR5, Casp8, Bid, Bcl2, and BclXL.

In this study, we showed that the antioxidant defense system is initiated in U87MG cells upon chaetocin treatment. Chaetocin led to ROS generation and upregulation of Nrf2^63^ target antioxidant gene expression namely *HMOX1, NQO1, GCLM*, and *TNXRD1*^64,65^. We also interrogated the functional role of one of these, HMOX1, in apoptotic response and showed that genetic ablation of *HMOX1* further sensitized cells to apoptosis. Also, high HMOX1 expression was negatively correlated with patient survival. Since there are multiple antioxidant genes, effect of a single gene might not broadly represent the correlation between antioxidant defense mechanism and patient survival. Therefore, analyzing the effects of multiple antioxidant defense genes will be critical. We show that chaetocin mediated GBM cell sensitization to TRAIL, FasL, and BH3 mimetics is ROS dependent, supported by several studies showing the interplay between TRAIL-mediated signaling and oxidative stress responses. For example, ROS production was previously shown to upregulate DR5 expression in human carcinoma cell lines^66^. Baicalein^67^ and Vitisin A^68^ sensitized prostate cancer cells to TRAIL via ROS generation and DR5 upregulation. As another important modulator of oxidative stress response, Glutathione reductase inhibitors potentiated TRAIL toxicity in prostate carcinoma and melanoma^69^.

Chaetocin was illustrated to inhibit the progression of various cancer types including chronic myelogenous leukemia^70^ and non-small cell lung cancer^59^ through oxidative stress induction. Chaetocin was also shown to inhibit invasive ability and trigger cell cycle arrest of the human intrahepatic cholangiocarcinoma in ROS-dependent manner^71^. Our results showing the chaetocin-induced cell cycle arrest are in accordance with these findings. As another literature-supported finding, hallmark EMT pathway was negatively enriched upon chaetocin treatment in our GSEA results.

In glioma, chaetocin was previously shown to induce ROS-mediated apoptosis through the Atm–Yap1 axis and Jnk-dependent metabolic adaptation, where chaetocin reduced lactate levels, ATP production and glucose uptake^51^. In concordance, our GSEA results revealed oxidative phosphorylation and glycolysis as negatively enriched upon chaetocin treatment, implying metabolic rewiring of glioma cells by chaetocin treatment. In glioma, chaetocin mediated activation of JNK resulted in apoptosis via inhibition of Bcl2^72^, as well as activation of p53^73^, suggesting a similar mechanism as identified in our GBM work. p53 mediated TRAIL sensitization is likely linked to increased expression of p53 target genes such as DR5, *BAX, NOXA*, and *PUMA*^74^.

Although modulation of SUV39H1 activity can induce ER stress and subsequent apoptosis in lung cancer^59^, we show that chaetocin effects were independent of SUV39H1 regulation. Chaetocin contains the reactive epipolythiodioxopiperazine motif^29^ and apoptosis induction in GBM cells might be attributed to enhanced ROS production through covalent inhibition of TXNRD1^75,76^, thereby rendering the ROS defense thioredoxin system inactive.

Taken together, we postulate a model in which ROS production by chaetocin treatment increases the apoptotic priming of GBM cells and renders them more prone to apoptosis initiated by other intrinsic and extrinsic agents. Elevated cellular ROS levels cause DNA damage and p53 activation. Active p53 initiates DNA repair mechanisms and render mitochondria primed for Cytochrome C release and consequent apoptosis in case damage is unrepairable. To eradicate the detrimental effect of cellular ROS accumulation, antioxidant defense mechanisms get activated in response to chaetocin treatment. When chaetocin is combined with extrinsic and intrinsic apoptosis inducers, ROS mediated primed state of mitochondria, as well as elevated proapoptotic gene expression (*FADD, CASP3, CASP8, DR4, PUMA, NOXA, BAD, BIM*, and *HRK)* render cells much more prone to apoptosis (Fig. 8g).

Our identification of chaetocin as an apoptosis-sensitizer makes it a strong weapon against GBMs, and possibly a wide range of cancers. Importantly, previously revealed ability of chaetocin to cross blood brain barrier^51^, as well as our illustration on potency of chaetocin and TRAIL combination in reducing tumor growth in vivo offers a potential therapeutic approach against GBM.

## Supplementary information

Supplementary Info

Supplementary Video 1 Control (Chaetocin+TRAIL experiment)

Supplementary Video 2 Chaetocin (Chaetocin+TRAIL experiment)

Supplementary Video 3 TRAIL (Chaetocin+TRAIL experiment)

Supplementary Video 4 Chaetocin+TRAIL (Chaetocin+TRAIL experiment)

Supplementary Video 5 Control (Chaetocin+FasL experiment)

Supplementary Video 6 Chaetocin (Chaetocin+FasL experiment)

Supplementary Video 7 FasL (Chaetocin+FasL experiment)

Supplementary Video 8 Chaetocin + FasL (Chaetocin+FasL experiment)

Supplementary Video 9 Control (Chaetocin+ABT263 experiment)

Supplementary Video 10 Chaetocin (Chaetocin+ABT263 experiment)

Supplementary Video 11 ABT263 (Chaetocin+ABT263 experiment)

Supplementary Video 12 Chaetocin + ABT263 (Chaetocin+ABT263 experiment)

Supplementary Video 13 Control (Chaetocin+WEHI539 experiment)

Supplementary Video 14 Chaetocin (Chaetocin+WEHI539 experiment)

Supplementary Video 15 WEHI539 (Chaetocin+WEHI539 experiment)

Supplementary Video 16 Chaetocin + WEHI539 (Chaetocin+WEHI539 experiment)
