# Supplementary material for: The fungal metabolite chaetocin is a sensitizer for pro-apoptotic therapies in glioblastoma
Source: Cell Death Dis. 2019 Nov 26;10(12):894. doi: 10.1038/s41419-019-2107-y (PMC6879621; doi:10.1038/s41419-019-2107-y)
Supplement: Supplementary file 1 — Supplementary Info [file 41419_2019_2107_MOESM1_ESM.pdf]

SUPPLEMENTARY INFORMATION

1- SUPPLEMENTARY FIGURES

A

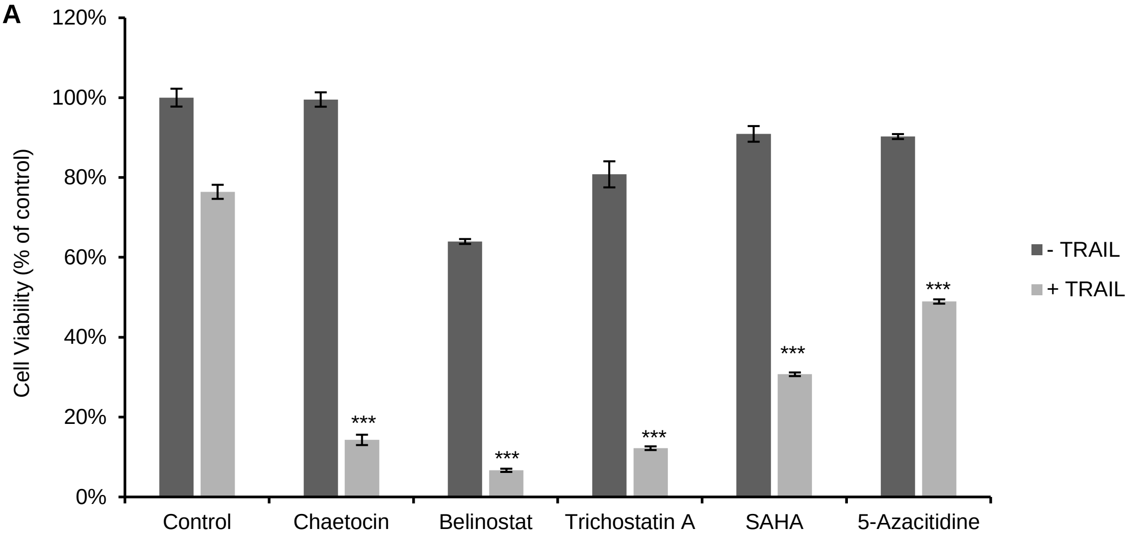

B

Dose Chae (nM)	Dose TRAIL (ng/ml)	Effect (% apoptotic cells)	CI Value
100.0	2.5	95	0.028
100.0	5.0	97	0.030
100.0	1.0	89	0.033
100.0	10.0	97	0.038
200.0	2.5	97	0.038
200.0	5.0	98	0.040
200.0	1.0	96	0.041
100.0	0.5	73	0.043
200.0	0.5	93	0.047
25.0	1.0	27	0.052
200.0	10.0	97	0.058
25.0	0.5	11	0.059
25.0	2.5	47	0.064
25.0	5.0	56	0.093
500.0	1.0	96	0.096
500.0	0.5	95	0.101
500.0	2.5	95	0.106
500.0	5.0	95	0.119
200.0	25.0	93	0.143
100.0	25.0	90	0.150
25.0	10.0	61	0.152
500.0	10.0	93	0.154
200.0	50.0	95	0.209
500.0	25.0	93	0.211
100.0	50.0	91	0.257
25.0	25.0	71	0.283
500.0	50.0	94	0.297
25.0	50.0	72	0.540

C

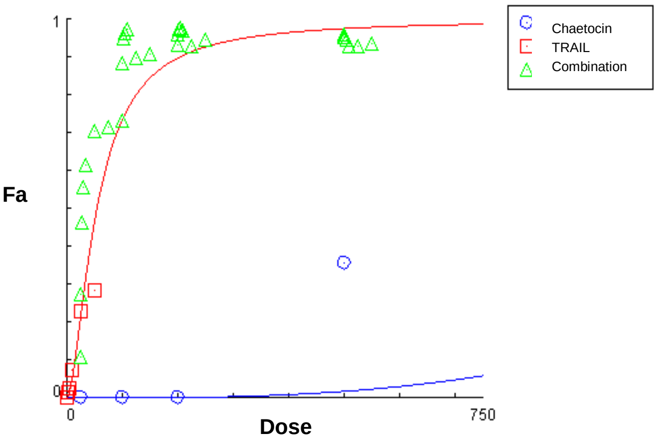

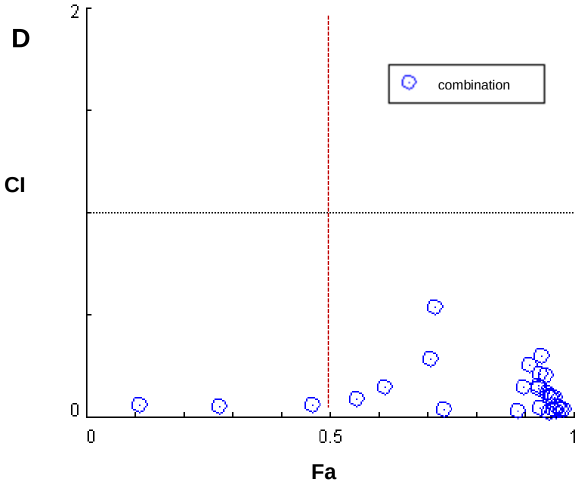

Supplementary Figure 1: (a) Viability analysis of U87MG cells upon Chaetocin (50 nM), Belinostat (5 μM), Trichostatin A (500 nM), SAHA (1 μM) and 5-Azacitidine (10 μM) treatments for 24 h followed by TRAIL (100 ng/ml, 24h) treatment. Data were normalized to untreated control. (*** denote $P<0.001$, two-tailed Student's *t*-test) **(b)** Percent cell death and Combination index (CI) values for Chaetocin and TRAIL combination treatment, calculated by CompuSyn software. **(c)** Dose-dependent changes in effect levels (Fa). **(d)** CI vs effect level (Fa) plots.

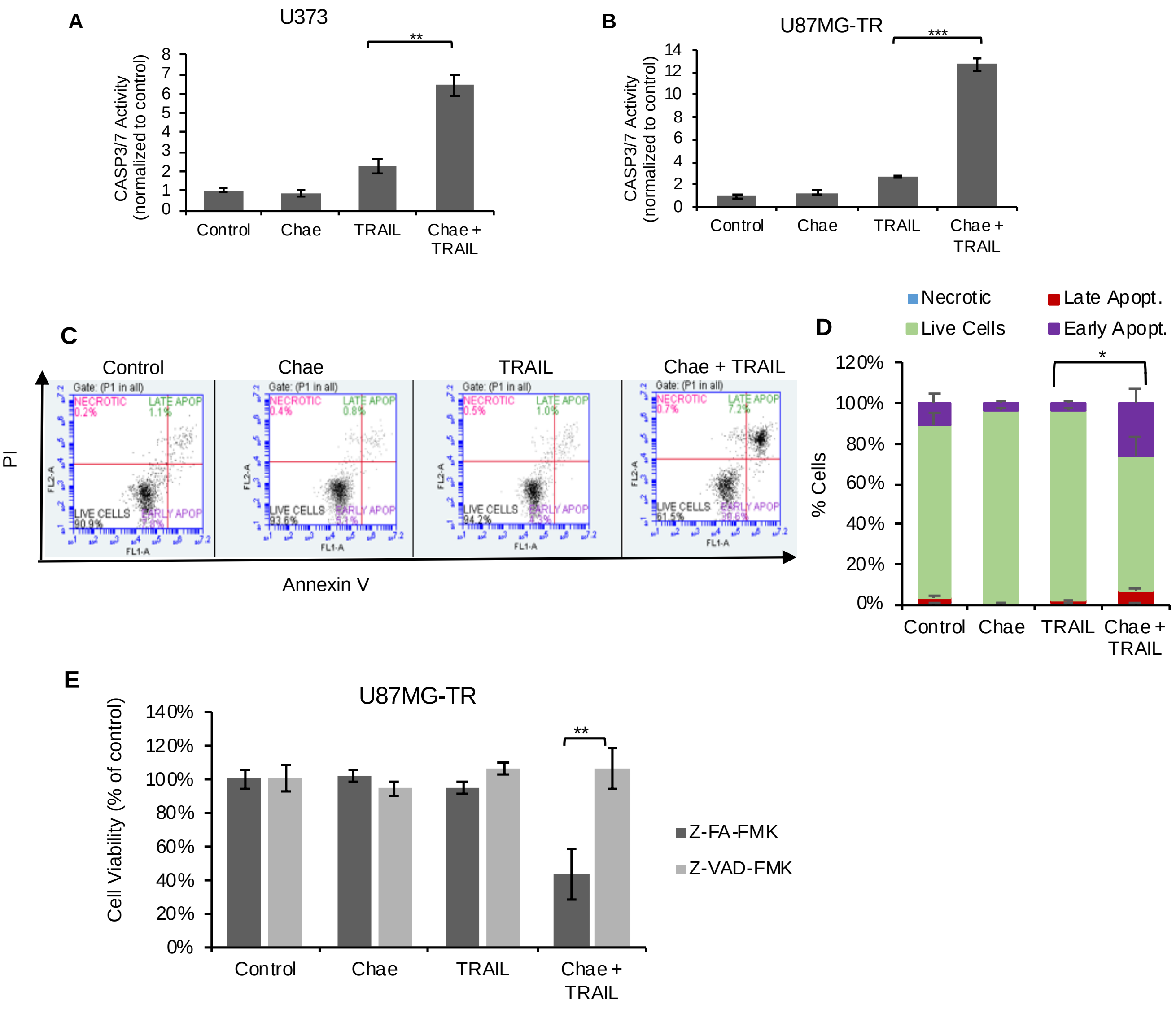

Supplementary Figure 2: (a) Caspase-3/7 activity of innately TRAIL resistant U373 cells upon Chaetocin (100 nM) and TRAIL (100 ng/ml) combination treatment. Data were normalized to untreated control cells. **(b)** Caspase-3/7 activity of U87MG cells with acquired TRAIL resistance (U87MG-TR) upon Chaetocin (100 nM) and TRAIL (100 ng/ml) combination treatment. Data were normalized to untreated control cells. **(c)** Flow cytometric analysis of AnnexinV/PI staining in U373 cells upon Chaetocin (100 nM) and TRAIL (100 ng/ml) combination treatment for 24h. (**d**) Quantification of flow cytometry data showing marked increase in apoptotic cell populations upon combination treatment. Data is normalized to total number of cells under each condition. **(e)** Viability analysis of U87MG-TR cells pretreated with caspase inhibitors (20 uM for 24h) followed by Chaetocin (100 nM) and TRAIL (100ng/ml) treatment for 24h in presence of inhibitors. Z-FA-FMK is negative control, Z-VAD-FMK is general caspase inhibitor. (*, ** and *** denote $P<0.05$, $P<0.01$ and $P<0.001$ respectively, two-tailed Student's *t*-test)

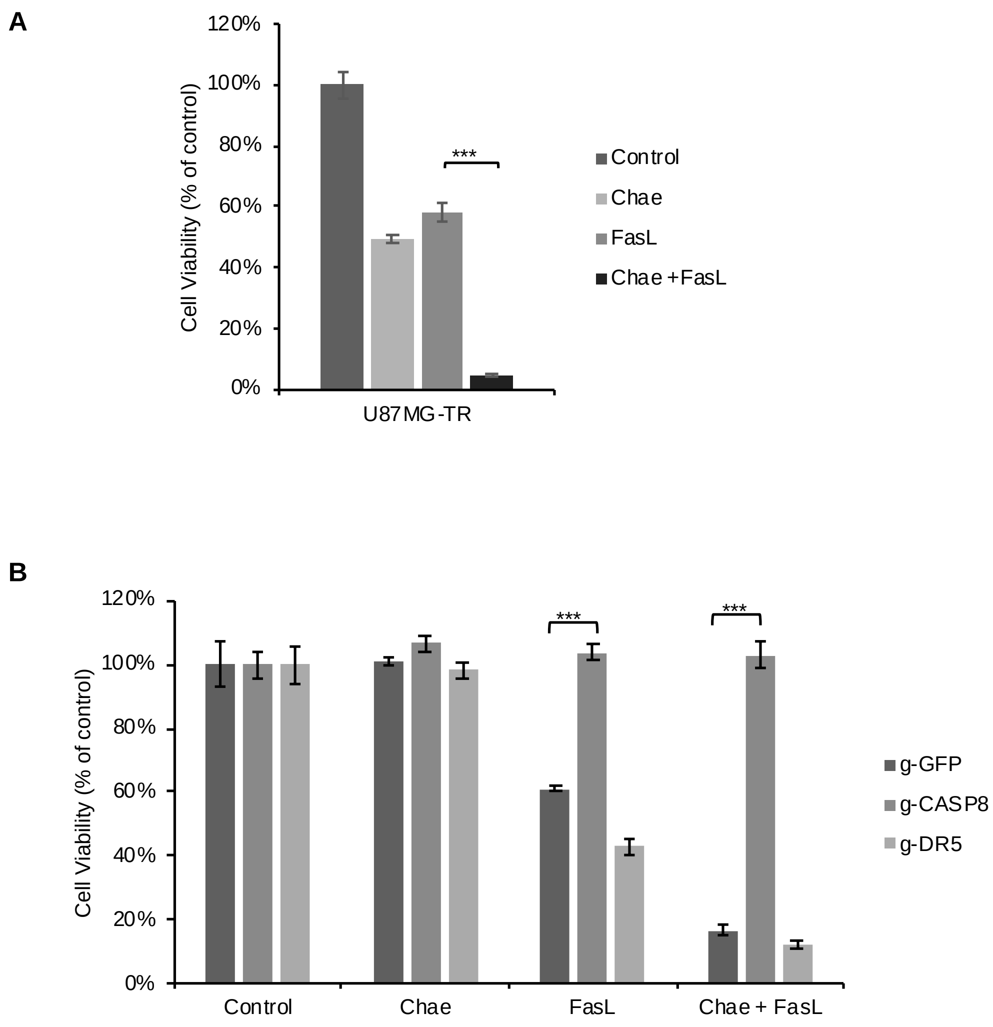

Supplementary Figure 3: (a) Viability analysis of U87MG-TR cells upon Chaetocin (100 nM) and FasL (100 ng/ml) treatment. Data were normalized to untreated control. **(b)** Viability analysis of CRISPR edited U87MG cells with Caspase 8 and DR5 knockouts upon combination treatment with Chaetocin (100 nM) and FasL (200 ng/ml) for 24h. Data were normalized to untreated control. (*** denotes *P*<0.001, two-tailed Student's *t*-test)

A

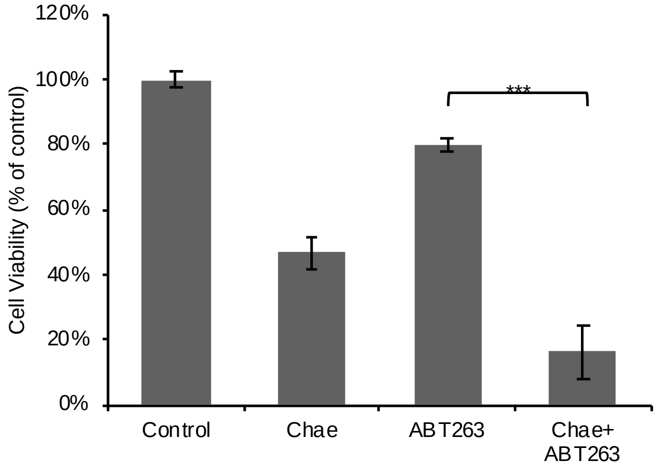

B

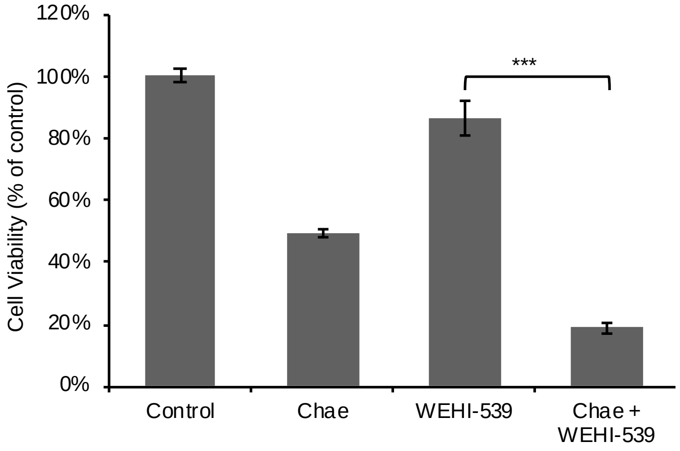

Supplementary Figure 4: (a) Viability analyses of GBM8 cells showing significantly reduced viability upon treatment with Chaetocin (100 nM) and ABT263 (1 μM) for 24h. **(b)** Viability analysis of GBM8 cells showing significantly reduced viability upon treatment with Chaetocin (100 nM) and WEHI539 (10 μM) for 24h (*, ** and *** denote $P<0.05$, $P<0.01$ and $P<0.001$ respectively, two-tailed Student's *t*-test)

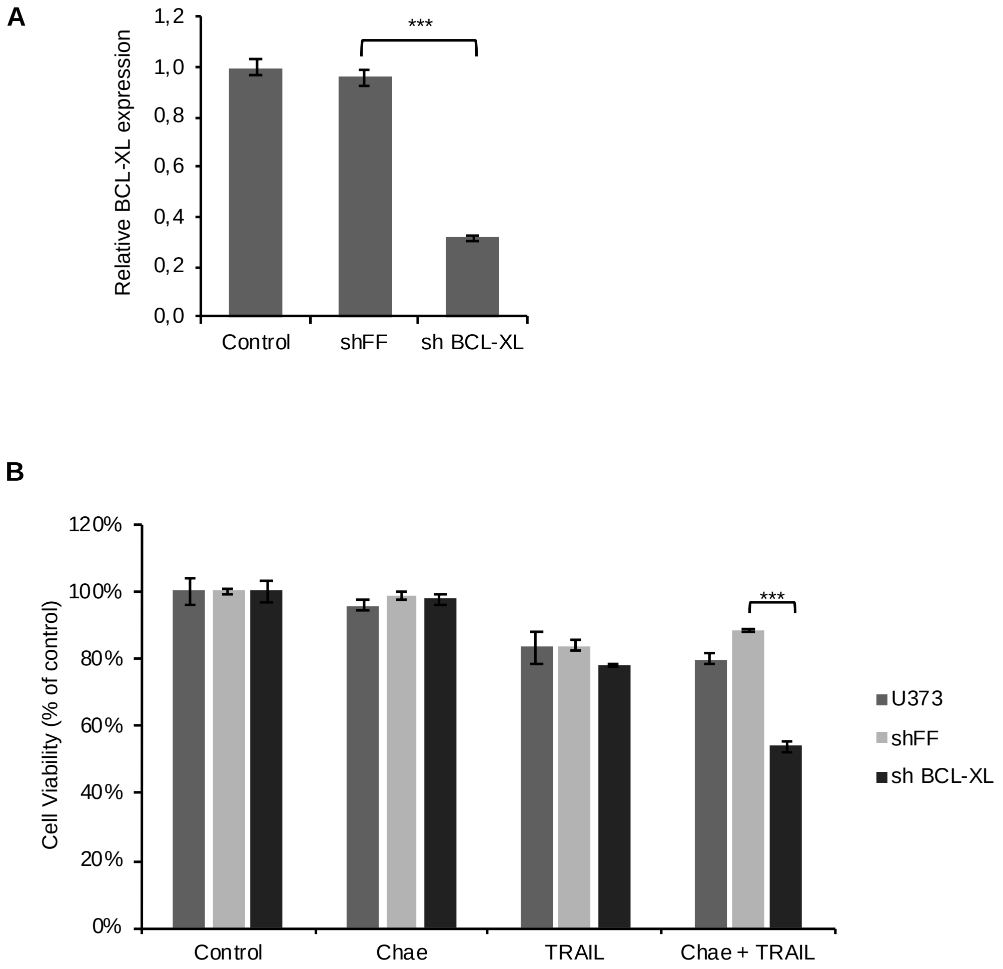

Supplementary Figure 5: (a) qPCR illustrating shRNA mediated knockdown of Bcl-XL in U373 cells. shFF is negative control shRNA. **(b)** Viability of U373 cells that are untransduced, shBclXL or shFF transduced, upon treatment with Chaetocin+ TRAIL. (*** denotes *P*<0.001, two-tailed Student's *t*-test)

A

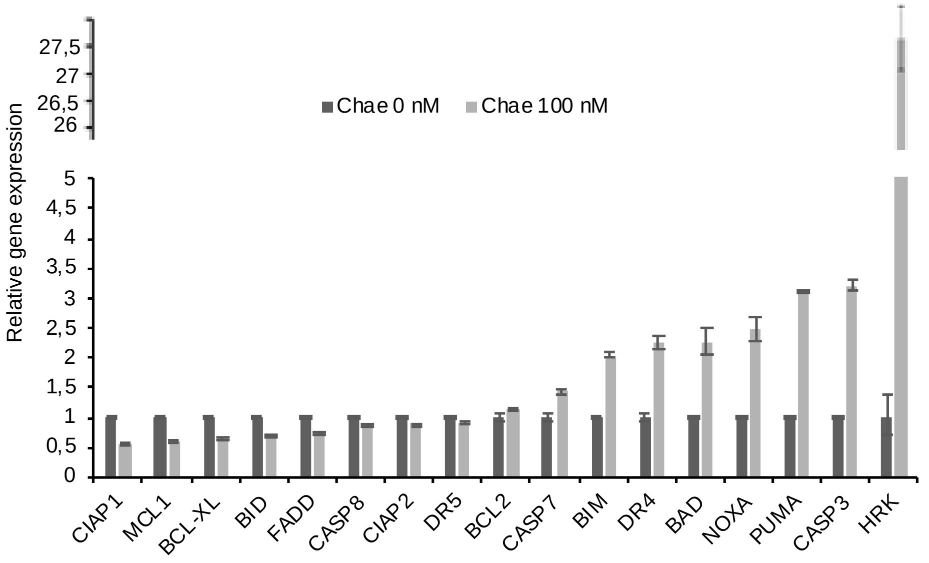

B

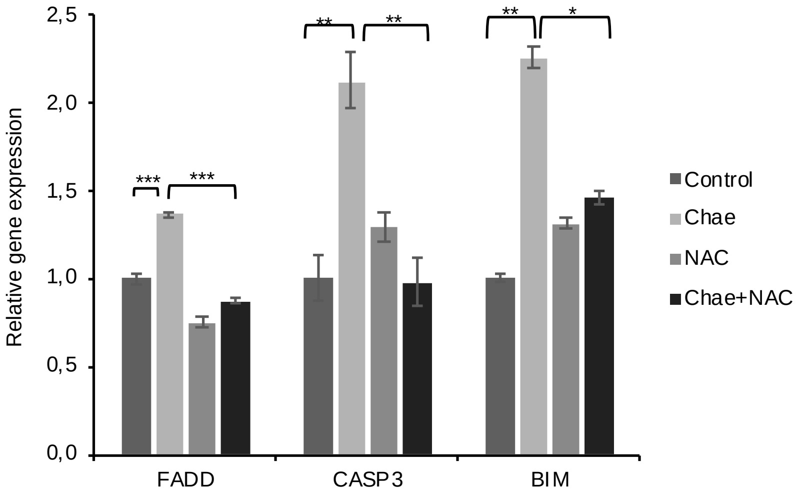

Supplementary Figure 6: (a) qPCR analysis of apoptosis related genes upon Chaetocin treatment (100 nM, 24h). Proapoptotic genes (*PUMA, NOXA, CASP3, HRK, BIM, BAD* and *DR4*) were upregulated by Chaetocin. Data were normalized to untreated control. **(b)** qPCR analysis demonstrating the effect of NAC on Chaetocin-induced changes in the expression of *FADD, CASP3* and *BIM*. NAC and Chaetocin were applied as 10 μM and 100 nM, respectively, for 24h. (*, ** and *** denotes P<0.05, P<0.01 and P<0.001 respectively, two-tailed Student's t-test)

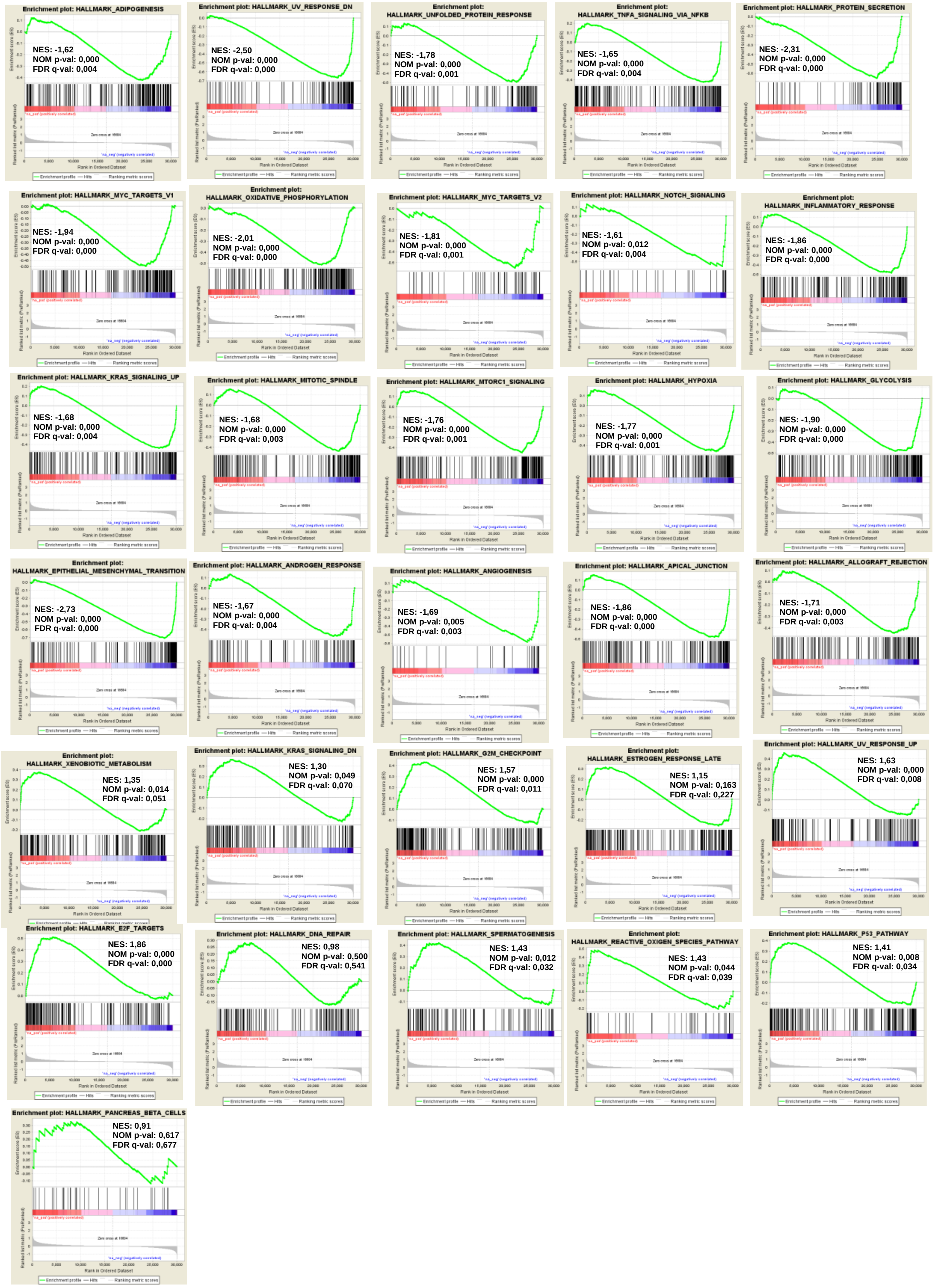

Supplementary Figure 7: Enrichment plots for hallmark pathways upon Chaetocin treatment (50 nM, 24h), obtained from GSEA. Normalized enrichment scores (NES), NOM p values and FDR q values were depicted on each graph.

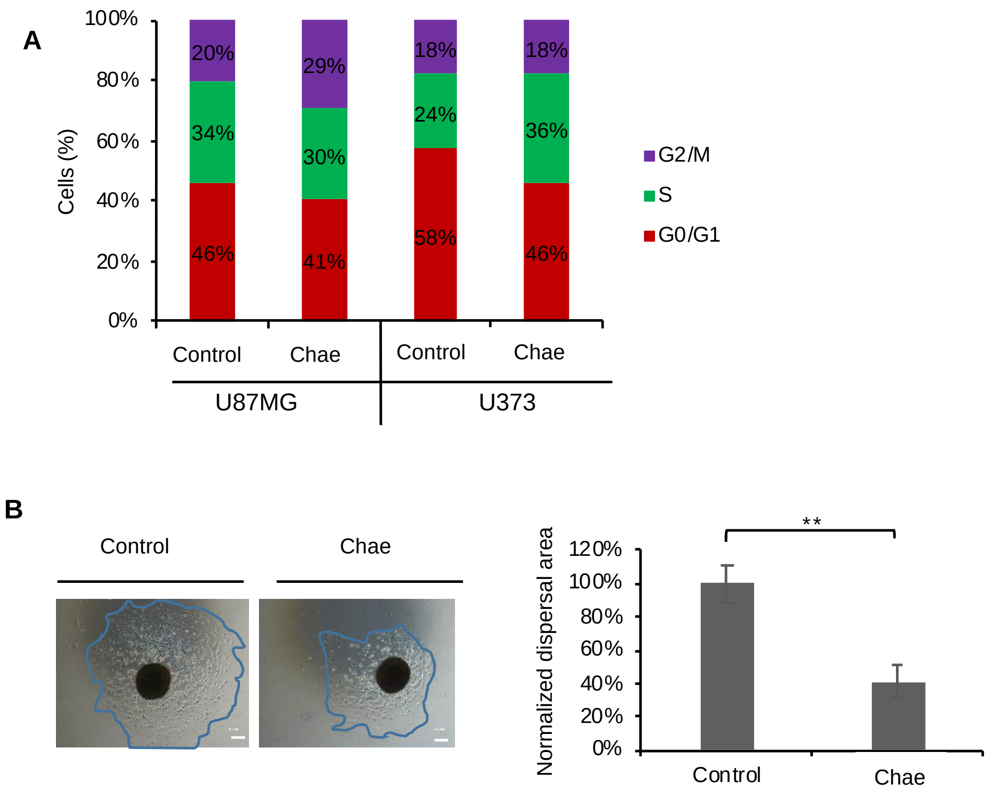

Supplementary Figure 8: (a) Flow cytometric analysis showing the effect of Chaetocin on cell cycle distribution of the U87MG and U373 cells. Percentage of cells in G2/M, S and G0/G1 phases was shown upon Chaetocin (100 nM) treatment for 24h. **(b)** *Left:* Representative images from spheroid invasion assays to measure the migration ability of GBM cells upon Chaetocin treatment (50 nM, 24h). Scale bar: 200 µm. *Right*: Quantification of dispersal area of spheroids. Data were normalized to untreated control. Images were taken by inverted live-cell light microscope (4x magnification). (** denotes P<0.01, two-tailed Student's *t*-test)

A

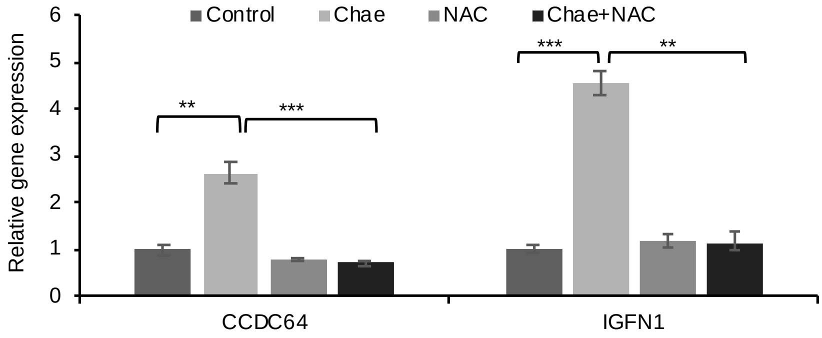

B

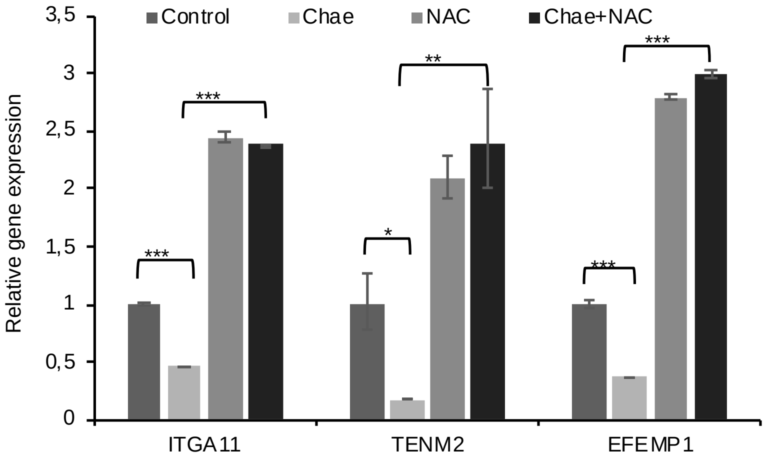

Supplementary Figure 9: (a) qPCR-based analysis of expression levels of selected upregulated (*CCDC64, IGFN1*) and **(b)** downregulated (*ITGA11, TENM2 AND EFEMP1*) genes obtained from RNAseq analysis. NAC and Chaetocin were used as 10 uM and 100 ng/ml, respectively, for 24h. Data were normalized to untreated control. (*, ** and *** denote *P*<0.05, *P*<0.01 and *P*<0.001, respectively, two-tailed Student's *t*-test)

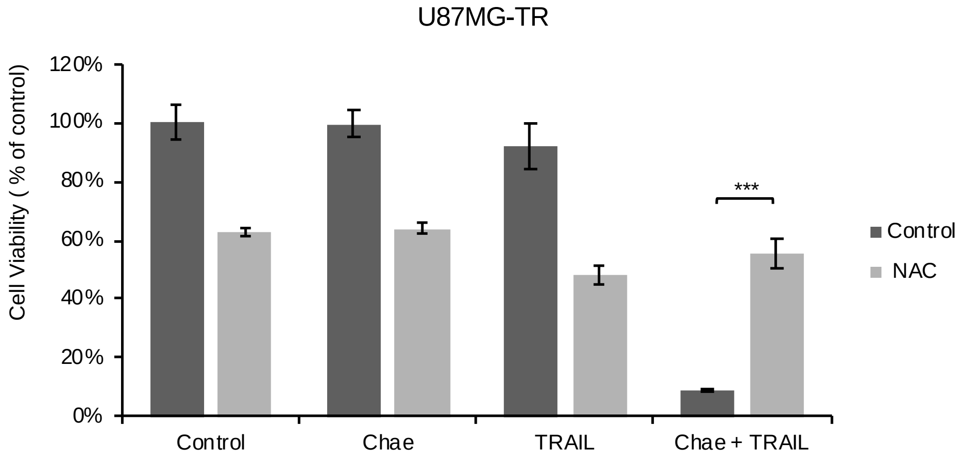

Supplementary Figure 10: Viability analysis of Chaetocin and TRAIL treated (100 nM, 100 ng/ml respectively for 24h) U87MG-TR cells in presence and absence of NAC (10 μM). Data were normalized to untreated control (*** denotes P<0.001, two-tailed Student's t-test)

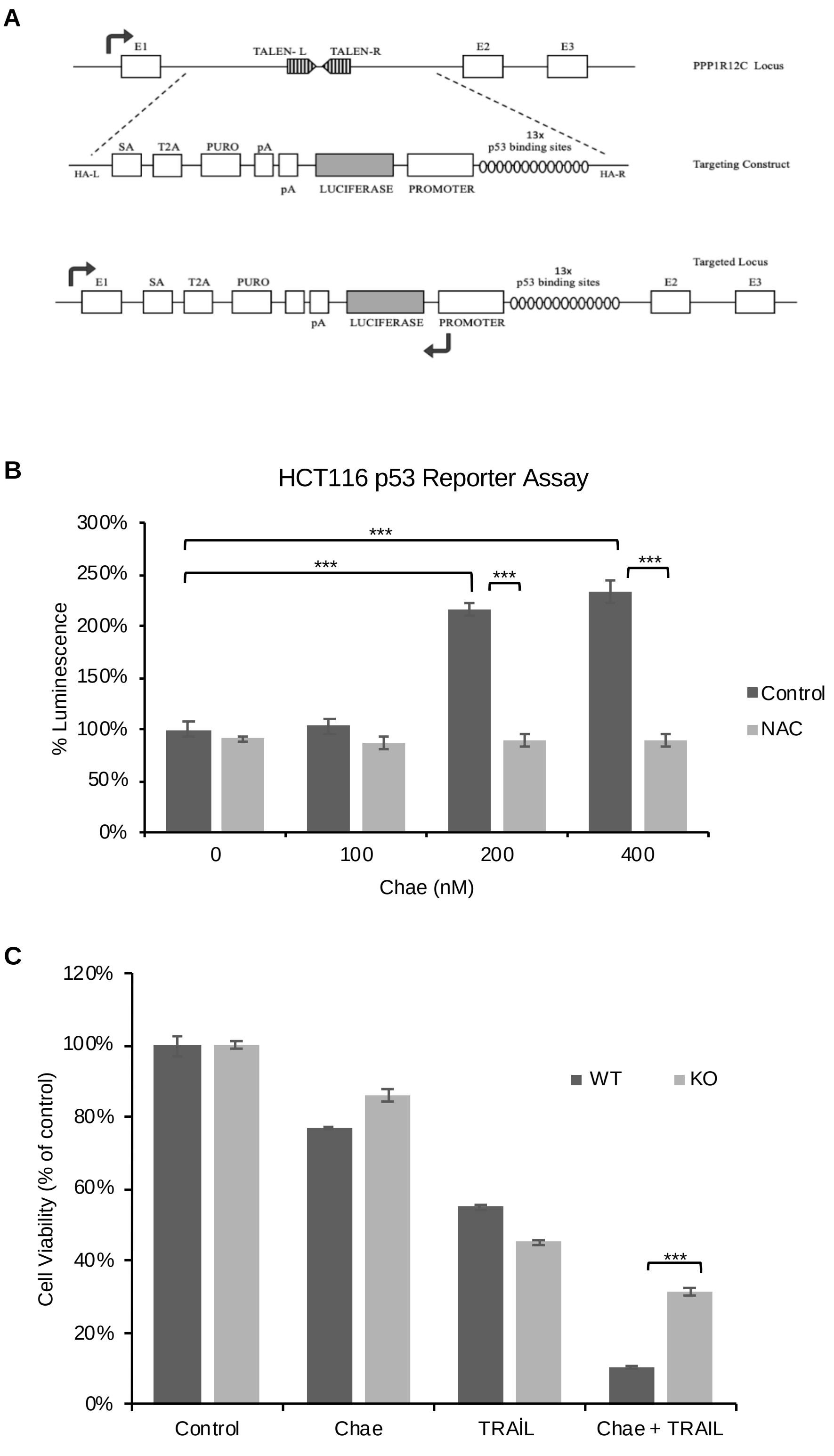

Supplementary Figure 11: (a) Schematic of the p53 reporter system **(b)** p53 transcriptional activity in HCT116 colon cancer cells, stably expressing luciferase under the p53 driven promoter, upon Chaetocin (100, 200, 400 nM for 24h) treatment. p53 transcriptional activity was induced in dose-dependent manner. NAC (10 μM) pretreatment was started 24h before Chaetocin (100 nM for 24 h) addition (**c)** Viability analysis of wild type (WT) and p53 knockout (KO) HCT116 cells upon Chaetocin and TRAIL treatment. Data were normalized to untreated control condition. (*** denotes *P*<0.001, two-tailed Student's *t*-test)

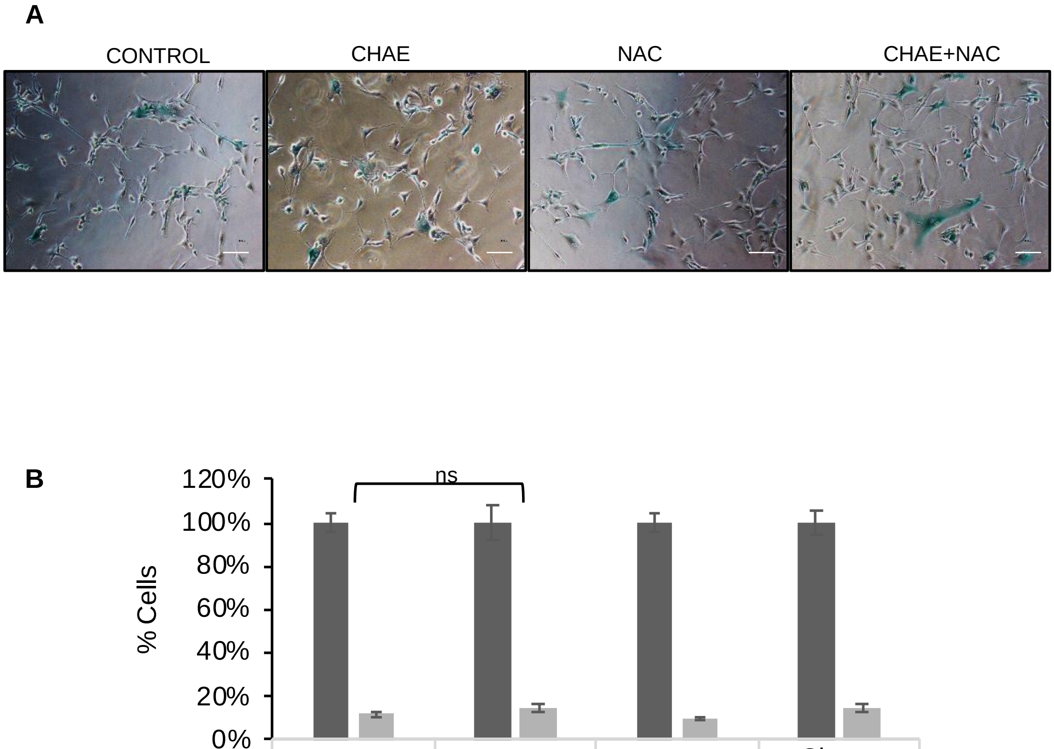

	Control	Chae	NAC	Chae+ NAC
TOTAL	100%	100%	100%	100%
Senescent	12%	15%	9%	15%

Supplementary Figure 12: (a) X-gal staining to show senescent cells upon Chaetocin treatment (100 nM, 24h) in ROS dependent manner (NAC pretreatment 10 μM, 24h). Images were taken by light microscope at 10x magnification. Scale bar: 200 μm **(b)** Quantification of X-gal staining showing no significant elevation in senescent state upon Chaetocin treatment. (ns denotes *P*>0.05, two-tailed Student's *t*-test)

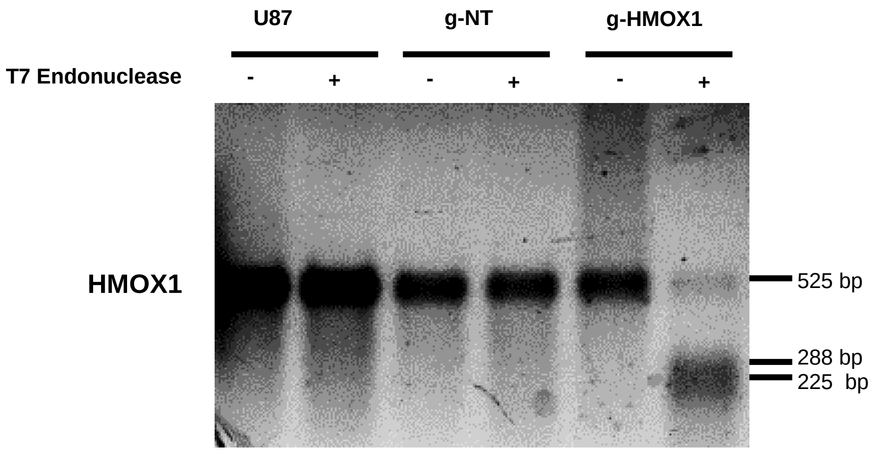

Supplementary Figure 13: T7 Endonuclease assay showing CRISPR knock out of HMOX1 gene. g-NT is negative control gRNA for CRISPR system.

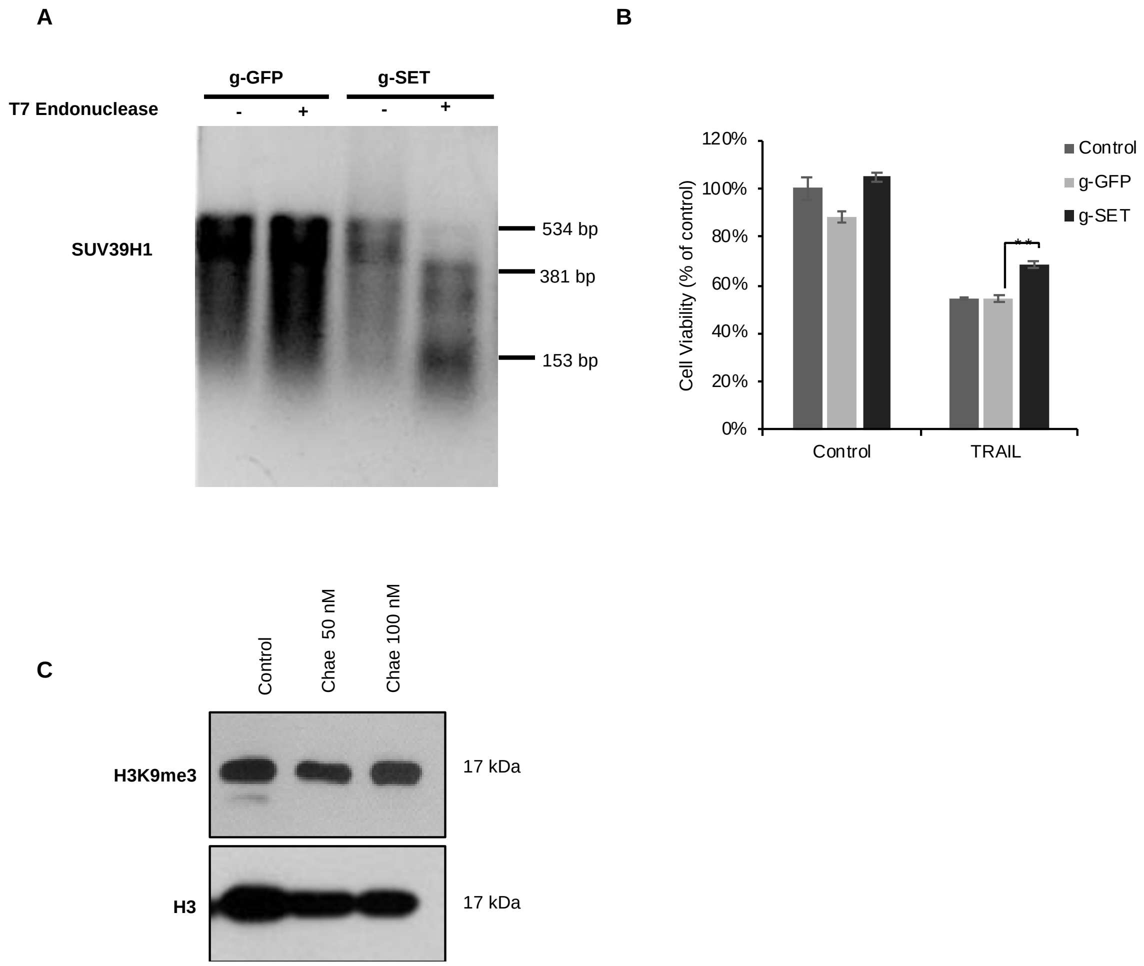

Supplementary Figure 14: (a) T7 endonuclease assay showing CRISPR mediated SUV39H1 knockout in U87MG cells. T2 is negative control gRNA for CRISPR assay **(b)** Viability analysis of U87MG cells with SUV39H1 knockout. Data were normalized to untreated control conditions. Depletion of SUV39H1 protein did not sensitize cells any further to apoptosis and rather rendered them slightly more resistant to TRAIL. (** denotes $P<0.01$, two-tailed Student's *t*-test). **(c)** Western blot analysis showing H3K9me(3) levels in Chaetocin (50,100 nM for 24h) treated cells.H3 was shown as loading control.

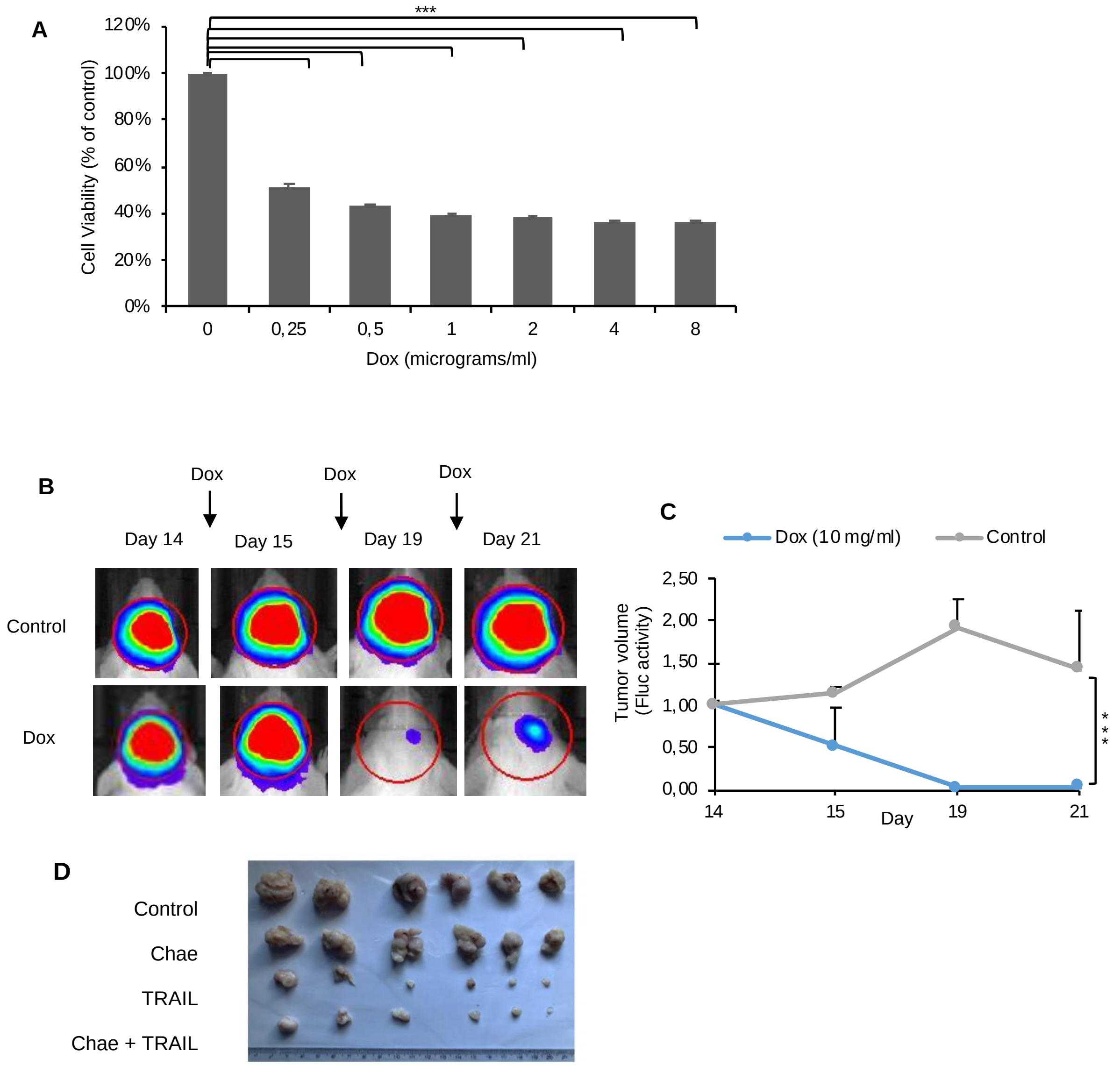

Supplementary Figure 15: (a) Viability analysis of Fluc-mCherry expressing and Tet-TRAIL transduced U87MG cells upon Doxycycline (Dox) treatment. Data were normalized to untreated control conditions. Increasing TRAIL secretion by elevating Dox concentration markedly reduced U87MG cell viability. (*** denotes $P<0.001$, two-tailed Student's *t*-test) **(b)** Representative images from intracranial tumors on day-14, day-15, day-16 and day-21 displaying normalized bioluminescent efficiencies acquired (blue to red indicates lower to higher radiance as photons/s/cm2/steradian). **(c)** Plots depicting tumor volumes of intracranial tumors under each condition (n=2/group). (*** denotes $P<0.001$, unpaired parametric *t*-test) **(d)** Representative subcutaneous tumors excised on day 30.

2- SUPPLEMENTARY TABLES

Supplementary Table 1. List of q-RT-PCR primers

Gene		Sequence
CHK2	F	CTCGGGAGTCGGATGTTGAG
	R	GAGTTTGGCATCGTGCTGGT
CHK1	F	CGGTATAATAATCGTGAGCG
	R	TTCCAAGGGTTGAGGTATGT
TP53BP1	F	CCTCAGGCTCTGGTGACTTC
	R	TGACAGCACAGCCCAGTAAG
BRCA2	F	CAGTGGTATGTGGGAGTTTGT
	R	ATCCATGACTTGCAGCTTCTC
H2AX	F	ACTCAACTCGGCAATCCAAG
	R	GGGTT AGCTGCAGAATTCCA
DDB2	F	CTCCTCAATGGAGGGAACAA
	R	GTG ACCACCATTCGGCTACT
EXO1	F	CTTTCTCAGTGCTCTAGTAAGGACTCT
	R	TGGAGGTCTGGTCACTTTGA
MSH2	F	GGAGAGATTGAATTTAGTGGAAGC
	R	TCATTTCCTGAAACTTGGAGAA
MSH6	F	CATGCGGCGACTGTTCTAT
	R	CACACTTCAGCAGGGACGTA
KU70	F	GCTAGAAGACCTGTTGCGGAA
	R	TGTTGAGCTTCAGCTTTAACCTG
RAD51	F	CTTTGGCCCACAACCCATTTC
	R	ATGGCCTTTCCTTCACCTCCAC
BRCA1	F	CTGAAGACTGCTCAGGGCTATC
	R	AGGGTAGCTGTTAGAAGGCTGG
MSH3	F	CCATCATGGCTCAGATTGGC
	R	ATCATGAGTGCTCGTCCCTC
MLH1	F	CTACTTCCAGCAACCCCAGA
	R	AGAACCTCATGTCCCTGCTC
PMS1	F	ATGAGTGGAGCAGGGGAAAT
	R	CACTTGCTGACATGGGTTTCT
PMS2	F	ACTTCCGTGGATTCTGAGGG
	R	GTGTTTGGGGTTGCGAGATT
MGMT	F	CCCGTTTTCCAGCAAGAGTC
	R	GGATTGCCTCTCATTGCTCC
ATM	F	CATCGCATGTGATTAAAGCA
	R	TTCTGATAGGAATCAGGGC
ATR	F	CAATTGTGGAGGAGATTTCC
	R	CTTCTGAGAACTCTTGTATCTG
BIM	F	GCAATGGCTTCCATGAGGCAG
	R	TCCACACCAGGCGGACAATG
CASP8	F	ACACCAGGCAGGGCTCAAAT
	R	GCAGGTTCATGTCATCATCCAGTT
CASP7	F	CGGTCCTCGTTTGTACCGTC
	R	CGCCCATACCTGTCACTTTATCA
CASP3	F	CATGGAAGCGAATCAATGGACT
	R	CTGTACCAGACCGAGATGTCA
Bad	F	CCCAGAGTTTGAGCCGAGTG
	R	CCCATCCCTTCGTCGTCCT
Bcl2	F	CAGGGCGATGTTGTCCACC

	R	GGGGAGGATTGTGGCCTTC
Bcl-XL	F	GGTCGCATTGTGGCCTTTTTC
	R	TGCTGCATTGTTCCCATAGAG
BIRC2 (CIAP1)	F	AGCACGATCTTGTCAGATTGG
	R	GGCGGGGAAAGTTGAATATGTA
BIRC3 (CIAP2)	F	CAGGGCTCCTGGGTAGAACT
	R	CTACTCCGTCCAGACTCATGC
FADD	F	GCTGGCTCGTCAGCTCAAA
	R	ACTGTTGCGTTCTCCTTCTCT
Hrk	F	AGGTTGGTGAAAACCCTGTG
	R	GCATTGGGGTGTCTGTTTCT
Mcl1	F	TGCTTCGGAAACTGGACATCA
	R	TAGCCACAAAGGCACCAAAAG
Noxa	F	ACCAAGCCGGATTTGCGATT
	R	ACTTGCACTTGTTCCTCGTGG
Puma	F	GACCTCAACGCACAGTACGAG
	R	AGGAGTCCCATGATGAGATTGT
DR4	F	ACCTTCAAGTTTGTCGTCGTC
	R	AACTCTCCCAAAGGGCTATGT
DR5	F	AAGACCCTTGTGCTCGTTGT
	R	AGGTGGACACAATCCCTCTG
Bid	F	CCTACCCTAGAGACATGGAGAAG
	R	TTTCTGGCTAAGCTCCTCACG
HMOX1	F	AAGAGGCCAAGACTGCGTTCC
	R	GCAGAATCTTGCACTTTGTTGCTG
ARL14EPL	F	AATTGGACAGAAACAACTGCAACAA
	R	GATGAGCCTGCCACTTTTGTCA
MLC1	F	GTGCCATTTCCTGCTCGGGT
	R	GCACTTGGAAAGCAGGCCAC
CCDC64	F	CACGGGCTGTCCAGGAACTG
	R	ACAGCATCTTGATCTCGGCCTG
ANO8	F	CACCTCCCAGGAACGCCAGA
	R	TGGATGATCCCACGTGCTGC
ITGA11	F	GTGGCCAGGGTTCACGGA
	R	CGTCTCCCGTCTTCTGGTAGC
ITGA2	F	CTGCTGGTGTTAGCGCTCAGT
	R	GGGTGAACCAACCAGTAACCAGT
TENM2	F	ACCAGCATCTTGGAGTTACGAAATA
	R	GTCCCTGCCACAACTTCCGA
EFEMP1	F	AACTCTGCTAGCTCAAGATTCACA
	R	TGCTGTCTCACAGGATCCCA
IGFN1	F	ACTATGGCAGGAAAGCTCCG
	R	TCGCCTGTCAGCTTGTTGAT
TrxR1	F	ACG AAA GGC AAG AAC GGC GA
	R	TCTTTACCTCAGTACAGCGTGTG
MYT1	F	AGGGTCCCTCAATGGCTCGT
	R	CCTGACAAACTCCGGTGGGT
NQO1	F	AGGCTGGTTTGAGCGAGTGTT
	R	ATGCCACTCTGAATTGGCCAGAG
GCLM	F	GCAGACGGGGAACCTGCTGAA
	R	ACATCTGGAAACTCCCTGACCAAAT

Supplementary Table 2. Antibodies used for Western blot and immunostaining experiments

Primary Antibodies	Purchased from
Cleaved Caspase-3	Cell Signaling/9664
Caspase-3	Cell Signaling/9665
Caspase-7	Cell Signaling/12827
Caspase 8	Enzo / ALX-804-242
Caspase-9	Cell Signaling/9508
PARP	Cell Signaling/9542
cleaved PARP	Cell Signaling/9541
Bim	Cell Signaling/2933
BID (Human specific)	Cell Signaling/2002
Alpha tubulin	Abcam /ab15246
Alpha tubulin	Sigma/T9026
Bcl-2 (human)	BD Biosciences/551098
Bcl-xL (human)	BD Biosciences/610746
DR5	ProSci/2019
p53	Santa Cruz/sc-126 L2217
H3K9me(3)	Abcam/ AB8898
H3	Cell Signaling / D1H2 4499S
H2AX(Ser139)	Sigma-Aldrich/ 05-636
**Secondary Antibodies**	**Purchased from**
Anti-rabbit	Santa Cruz/sc-2054
Anti-mouse	Santa Cruz/sc-2055

Supplementary Table 3. Guide RNA sequences for CRISPR

Gene		Sequence
DR5	F	CACCGATAGTCCTGTCCATATTTGC
	R	AAACGCAAATATGGACAGGACTATC
CASP8	F	CACCGTCCTTTGCGGAATGTAGTCC
	R	AAACGGACTACATTCCGCAAAGGAC
CASP3	F	CACCGAATGGACTCTGGAATATCCC
	R	AAACGGGATATTCCAGAGTCCATTC
CASP7	F	CACCGTTGATATTTAGGCTTGCCGA
	R	AAACTCGGCAAGCCTAAATATCAAC
BID	F	CACCGAGAACCTACGCACCTACGTG
	R	AAACCACGTAGGTGCGTAGGTTCTC
HMOX1	F	CACCGAAGGGCCAGGTGACCCGAGA
	R	AAACTCTCGGGTCACCTGGCCCTTC
SUV39H1_SET	F	CACCGAGCTTCGTCATGGAGTACGT
	R	AAACACGTACTCCATGACGAAGCTC
g-NT	F	CACCGACGGAGGCTAAGCGTCGCAA
	R	AAACTTGCGACGCTTAGCCTCCGTC
g-GFP-1	F	CACCGTGAACCGCATCGAGCTGAA
	R	AAACTTCAGCTCGATGCGGTTCAC
g-GFP-2	F	CACCGGAGCGCACCATCTTCTTCA
	R	AAACTGAAGAAGATGGTGCGCTCC

Supplementary Table 4. Sequences of shRNAs

shBcl-XL:

TGCTGTTGACAGTGAGCGAGCTCACTCTTCAGTCGGAAATTAGTGAAGCCACAGATGTA
ATTTCCGACTGAAGAGTGAGCCTGCCTACTGCCTCGGA

shBcl-2:

TGCTGTTGACAGTGAGCGAGGAGATAGTGATGAAGTACATTAGTGAAGCCACAGATGTA
ATGTACTTCATCACTATCTCCCTGCCTACTGCCTCGGA

Supplementary Table 5. PCR primers for T7 assay

Gene		Sequence
SUV39H1	F	CTGGGACGCATCACTGTAGA
	R	GATCAGTCTCCCAGGCCTTTC
HMOX1	F	GAGAACGTGGCCTGAATGAG
	F	ACAAAATGCCCAACATGGAACC

Supplementary Table 6. List of vectors used for the study.

Vectors	Cat. No
pUMVC	Addgene Plasmid #8449
pCMV-dR8.2 dvpr	Addgene Plasmid #8455
pCMV-VSV-G	Addgene Plasmid #8454
3544 pMIG Bcl-2	Addgene Plasmid #8793
3541 pMIG Bcl-XL	Addgene Plasmid #8790
pBABE GFP-puro	Addgene Plasmid #10668
MSCV	Addgene Plasmid #24828
Pico2-Fluc.mCherry	gift from Dr. Andrew Kung (Dana Farber Cancer Institute)
lentiCas9-Blast	Addgene Plasmid #52962
lentiCRISPR v2	Addgene Plasmid #52961
lentiGuide-Puro	Addgene Plasmid #52963
hAAVS1 1L TALEN	Addgene Plasmid #35431
hAAVS1 1R TALEN	Addgene Plasmid #35432
AAVS1 SA-2A-puro-pA donor	Addgene Plasmid #22075
PG13-luc (wt p53 binding sites)	Addgene Plasmid #16442
pGL3-Basic	Promega
pENTR1A	Addgene plasmid # 17398
pLIX_402	Addgene plasmid # 41394

3- SUPPLEMENTARY VIDEO LEGENDS

Supplementary Video 1-4. Live-cell imaging of U87MG cells upon Chaetocin (100 nM) and/or TRAIL (100 ng/ml) treatment for 16h. Experiments were carried out by Olympus Xcellence Pro inverted microscope (Center Valley, PA, USA) with a 10x air objective. Time-lapse images were captured right after drug treatments with 6-minute time intervals.

Supplementary Video 5-8. Live cell imaging of U87MG cells upon Chaetocin (100 nM) and/or FasL (200 ng/ml) treatment for 10h. Experiments were carried out by Olympus Xcellence Pro inverted microscope (Center Valley, PA, USA) with 10x air objective. Time-lapse images were captured right after drug treatments with 5-minute time intervals.

Supplementary Video 9-12. Live cell imaging of U87MG cells upon Chaetocin (100 nM) and/or ABT263 (1 μM) treatment for 24h. Experiments were carried out by Olympus Xcellence Pro inverted microscope (Center Valley, PA, USA) with 10x air objective. Time-lapse images were captured right after drug treatments with 5-minute time intervals.

Supplementary Video 13-16. Live cell imaging of U87MG cells upon Chaetocin (100 nM) and/or WEHI-539(1 μM) treatment for 24h. Experiments were carried out by Olympus Xcellence Pro inverted microscope (Center Valley, PA, USA) with 10x air objective. Time-lapse images were captured right after drug treatments with 5-minute time intervals.

4- SUPPLEMENTARY MATERIALS AND METHODS

Drug synergism calculation

U87MG cells were treated with multiple concentrations of TRAIL and Chaetocin and cell viability was calculated. CompuSyn software (Chou, 2010) based on Chou-Talalay model (Chou & Talalay, 1984) was used for synergy quantification. Dosage and the effect (decrease in cell viability) of both single drugs and the combination were given as an input to CompuSyn software which yields combination index values (CI) as an output. In Chou-Talalay model CI<1, =1, and >1 indicates synergistic, additive and antagonistic effect respectively.

RNAseq Library Preparation

Library preparation was performed by the Functional Genomics Laboratory (FGL), a QB3-Berkeley Core Research Facility at UC Berkeley. mRNA enrichment was performed on total RNA using polyA selection with the Invitrogen Dynabeads mRNA Direct kit. Subsequent library preparation steps of enzymatic fragmentation, adapter ligation and cDNA synthesis were done on the enriched RNA on an Apollo 324™ liquid handling system, with PrepX™ RNAseq Library Prep Kits (WaferGen/now TakaraBio). 15 cycles of PCR amplification was used for index addition and library fragment enrichment. Libraries were sequenced on an Illumina HiSeq4000 by the Vincent J. Coates Genomics Sequencing Laboratory at UC Berkeley, supported by NIH S10 OD018174 Instrumentation Grant.

Sphere Invasion Assay

Spheroids were generated by culturing 20.000 U87MG cells per sphere in 20 μl medium as hanging droplets on tissue culture plate lid for 3 days. The plate was filled with culture media to prevent drying of droplets due to evaporation. Generated spheres were transferred to 6 well plates by pipetting and after attaching to surface of the plate, spheres were treated with Chaetocin (100 nM) for 2 days. Number of the cells invading out of the spheres were counted by ImageJ software (NIH Image, NIH Bethesda, USA).

Cell Cycle Assay

Cells were seeded to 6-well plates (300.000 cells/well). After treatment with Chaetocin (100 nM for 24h), all cells (both live cells attached to culture plate and dead cells free floating in medium) were harvested and pelleted. Harvested cells were washed with PBS and then fixed with ice cold 70% ethanol by adding 1 ml ethanol dropwise to the pellet while gently vortexing and then incubating at 4°C for 30 minutes. Pellets were washed 2 times with PBS, spun at 850g, supernatant was carefully removed after each round. Pellets were treated with RNase

(50 µl of 100 µg/ml stock) and incubated for 15 min. 200 µl PI (from 50 µg/ml stock) was added and cells were incubated at RT for 30 minutes. Tubes were stored at 4°C, protected from light. Stained cells were analyzed by BD Accuri C6 (BD Biosciences, USA) flow cytometer (excitation 488 nm, emission 530/575 nm).

Xgal Staining

Cells were seeded to 6-well plates (300.000 cells/well) and treated with Chaetocin (100 nM for 24h). NAC treatment (10 µM) was applied 24h prior to and during Chaetocin treatment. After treatment, cells were washed with PBS and fixed with 2% formaldehyde, 0.2% glutaraldehye for 5 min at RT. Fixed cells were washed three times with PBS and stained with freshly prepared β-Gal staining solution (1 mg/ml X-Gal, 150 mM NaCl, 2 mM $MgCl_2$, 5 mM $K_3Fe(CN)_6$, 5 mM $K_4Fe(CN)_6$, 40 mM citric acid/sodium phosphate with pH 6.0) at 37 ˚C until blue color develops in positive control samples. X-gal solution was removed and cells were washed with PBS. Slides were covered with 50% Glycerol solution and stored at 4˚C. Images were taken by Nikon Eclipse TS100 Inverted Fluorescence Microscope (Nikon Instruments Inc.,NY,USA)

T7 Endonuclease assay

CRISPR edited cells were pelleted and their genomic DNA were isolated with MN Nucleospin Tissue kit (Macherey-Nagel, Germany). 30 cycle PCR was performed using specific surveyor primers (**Supplementary Table 5**) for each gene with following steps: initial denaturation 95°C for 3 min, denaturation 95 ^{o}C for 30 sec, annealing at 60 ^{o}C for 30 sec, extension 72 ^{o}C for 1 min and final extension 72 ^{o}C for 5 min. Amplified DNA were cleaned up with MN Gel and PCR extraction kit (Macherey-Nagel, Germany). T7 endonuclease digestion was performed as following steps: 95 ^{o}C initial denaturation, (-2 ^{o}C/sec) 95 ^{o}C → 85 ^{o}C, (-0.1 ^{o}C /sec) 85 ^{o}C →25 ^{o}C. 1µl T7 endonuclease enzyme was added and samples were incubated at 37 ^{o}C for 2 hours. Reaction was stopped by adding 1.5 µl 0.25 M EDTA to each sample. Samples were run on 1.5 % gel and visualized.

Histone extraction

Cells were harvested and washed twice with ice-cold PBS. Cells were resuspended in Triton Extraction Buffer (TEB: PBS containing 0.5% Triton X 100 (v/v), 2 mM phenylmethylsulfonyl fluoride (PMSF), 0.02% (w/v) NaN3) at a cell density of 10^7 cells per ml. Cells were lysed on ice for 10 minutes with gentle stirring and then centrifuged at 6,500 x g for 10 minutes at 4°C to spin down the nuclei. Supernatant was discarded. Nuclei was washed in half the volume of TEB and centrifuged as before. The pellet was resuspended in 0.2 N HCl. Histones were acid extracted over night at 4°C. 1M NaOH was added as of 1/5 volume of the HCl solution.

Samples were centrifuged at 6,500 x g for 10 minutes at 4°C to pellet debris. The supernatant which contains the histone proteins was stored. Protein content was quantified by Pierce BCA Protein Assay Kit (Cat.No:23227, Thermo Scientific,USA).

Luciferase reporter cell lines

A stable p53 reporter cell line was generated in HCT116 human colon cancer cells by inserting a single copy donor DNA into the hAAVS safe harbor site. The donor DNA plasmid was constructed by assembling DNA fragments containing 13 p53 binding sites and the Polyoma Virus promoter (from the PG13-luc plasmid), luciferase gene (from the pGL3 plasmid) into the AAVS1 SA-2A-puro-pA plasmid. This donor DNA was transfected into HCT116 cells along with two TALEN plasmids targeting a safe harbor site (hAAVS1 1L TALEN and hAAVS1 1R TALEN) using a 1:3 ratio (w/w) of plasmid: Polyethylenimine (PEI - Polysciences 23966). Stable transfectant colonies were selected with 1 μg/ml Puromycin (Sigma P9620) for 14 days and screened by PCR for correct integration. A highly p53 responsive colony was selected by treatment with 1μM Doxorubicin (Sigma D1515) followed by luciferase assays.

Luciferin reporter assay

HCT116 cells stably expressing luciferase under the p53 driven promoter were seeded to 96 well plate as 5.000 cells/well. NAC (10 μM) pretreatment started 24h before Chaetocin (100, 200, 400 nM for 24h) treatment. After treatment, 100 μg/ml D-Luciferin was added to each well and incubated for 10 min. Bioluminescence was measured by Synergy H1 plate reader (BioTek, VT, USA) and Gen5 data analysis software.

In Vivo Tumor Growth with Doxycycline Inducible TRAIL Expression

Non-obese diabetic/severe combined immunodeficiency (NOD/SCID) mice housed and cared in appropriate conditions of Koç University Animal Facility were used and all protocols were approved by the institution boards of Koç University. Firefly Luciferase (Fluc) and mCherry expressing stable U87MG were transduced with Tet-TRAIL viruses. 1x 10^5 cells were injected in 7μl PBS intracranially using stereotaxic injection (Coordinates: 2 mm lateral, 2 mm caudal; 2 mm deep from bregma). Presence and progression of tumors was monitored by repeated noninvasive bioluminescence imaging (IVIS Lumina III). Accordingly, mice were injected with 150 μg/g body weight of D-Luciferin intraperitoneally and sum of the photon counts of tumor regions were obtained. To test the effect of Doxycycline (Dox) treatment induced TRAIL expression on tumor growth, mice with established tumors were treated with either saline or Dox (10 mg/ml) as intraperitoneal injections (once in every three-days starting from day 14).

Quantification of tumor progression was performed with GraphPad PRISM software (San Diego, CA, USA)
